# The Prevalence of Sexual Assault Among Higher Education Students: A Systematic Review With Meta-Analyses

**DOI:** 10.1177/15248380231196119

**Published:** 2023-09-20

**Authors:** Bridget Steele, Mackenzie Martin, Alessandra Sciarra, G. J. Melendez-Torres, Michelle Degli Esposti, David K. Humphreys

**Affiliations:** 1 University of Oxford, UK; 2 University of Exeter, UK

**Keywords:** sexual violence, sexual assault, higher education, prevalence, review, meta-analysis

## Abstract

Sexual assault among higher education students has detrimental impacts on the health and educational outcomes of survivors. This systematic review aims to describe and synthesize the available quantitative evidence on sexual assault prevalence among this population. We searched Medline, EMBASE, Global Health, PsycINFO, Web of Science, ERIC, and CINAHL for studies published in English, French, Italian, and Spanish from database inception to August 2020 (updated May 2022). We screened studies using prespecified inclusion criteria for the population and context (registered higher education students), condition (self-reported sexual assault), and study design (quantitative survey). The Joanna Briggs Institute Critical Appraisal Checklist was used to assess study quality. Prevalence estimates disaggregated by type of sexual assault, gender identity, and world region were meta-analyzed using a random-effects model and reported following PRISMA guidance. We identified 131 articles, from 21 different countries. The meta-analyzed prevalence of sexual assault was 17.5% for women, 7.8% for men, and 18.1% for transgender and gender diverse people. Four types of sexual assault were identified: rape, attempted rape, forced sexual touching, and coercive sex. Forced sexual touching was the most common act experienced. The African Region had the highest prevalence estimates for women’s sexual assault, and the Western Pacific region had the highest prevalence estimates for men’s sexual assault. Higher education institutions, especially those outside of the United States, should commit to the implementation of surveys to monitor sexual assault prevalence and dedicate increased resources to supporting student survivors of sexual assault.

## Introduction

Sexual assault (SA), defined as any unwanted attempted or completed physical contact that is sexual in nature, experienced by higher education (HE) students is a public health and human rights issue (DeMatteo et al., 2015; DeVore & Sachs, 2011; Mellins et al., 2017). Experiencing SA as an HE student can result in significant consequences for survivors’ physical health and mental well-being (Black et al., 2016; Krebs et al., 2007; McMahon et al., 2021) as well as educational outcomes (Baker et al., 2016). Immediate health concerns for survivors can include unwanted pregnancy and sexually transmitted infections (Jina et al., 2010). In the long term, SA survivors are more likely to engage in risky sexual behaviors (nine times more likely) (Howard & Wang, 2005), develop disordered eating (five more likely) (Ackard & Neumark-Sztainer, 2002), suffer from poor mental health (three times more likely) (Agardh et al., 2012), and consider suicide (two times more likely) (Howard & Wang, 2005). Gastrointestinal issues have also been associated with SA (Astbury, 2006; Leserman, 2005), with women survivors being two times more likely to report nausea, vomiting, abdominal pain, and diarrhea than women who had not experienced SA (Golding, 1994). Further chronic conditions such as pain, migraines, insomnia, and fatigue are prevalent conditions among survivors (Astbury, 2006; Green et al., 1999; Kimerling & Calhoun, 1994; Leserman, 2005). In addition to health outcomes, experiencing SA in HE can impact academic outcomes. A systematic review on SA and academic outcomes in university settings found that across 13 articles, SA was associated with lower grade point average, dropping out, and learning problems (Molstad et al., 2021). Further, a rigorous longitudinal study on SA in HE found that SA exposure correlated with lower grade point average (GPA) and that survivors of SA were two more times likely to leave HE without completing their degree (Baker et al., 2016).

The most common way SA is measured in HE is through campus climate surveys. Underreporting of SA means that formal reports to institutions or to law enforcement officials have limited utility and accuracy for estimating prevalence and informing intervention efforts, and campus climate surveys have the potential to ascertain more accurate prevalence rates (Cantalupo, 2014) (Sinozich & Langton, 2014). Campus climate surveys gather self-report data from students to help to understand the scope of SA in HE and to inform response and prevention efforts (Moylan et al., 2018). They can measure prevalence and risk factors and assess institutional climate (including students’ perceptions of leadership competence, beliefs about other students, or knowledge of policies and supports) (Bull et al., 2022; Krause et al., 2018; Moylan et al., 2018). Campus climate surveys emerged as a result of the American Task Force to Protect Students from SA, which provided federal-level guidance on how to prevent SA in HE (Not alone: The first report of the White House task force to protect students from sexual assault, 2014). While they are most popular in the United States, educational institutions around the world have begun to adopt and adapt them. As a result, there is now an abundance of studies, globally, estimating SA prevalence among HE students, but these data have yet to be systematically reviewed or meta-analyzed. It is important to include the evidence base outside of the United States in our understanding of SA prevalence. The vast majority of discussion surrounding prevention and response of SA among HE students is centered in the American context due to the leadership role U.S. academics and U.S. HE institutions have played in identifying and taking responsibility for responding to SA (Bull et al., 2022; Steele et al., 2022). However, SA is a global phenomenon, and different contextual factors such as economic and sociocultural factors, norms, and laws mean that we could expect differing rates of SA among HE students across geographical regions (García-Moreno et al., 2013). Further, the structure and role of HE varies globally. Equipping HE institutions and policy makers with regionally specific data prevalence is an important step for allowing prevention and response initiatives to be more contextually tailored.

Campus climate surveys have consistently found that women face the burden of SA when compared to men (Cantor et al., 2020). There is also increasing evidence that transgender and gender diverse students could face an increased risk of SA (Coulter et al., 2017). Therefore, it is important to measure, report on, and synthesize estimates by gender identity.

Reviews published in recent years have synthesized specific bodies of evidence on the prevalence of SA experienced by HE students. For example, a review of 22 studies on SA from U.S. colleges found that the prevalence of SA victimization for female students ranged from 4.7% to 58% (Stoner & Cramer, 2019). Another U.S. review included 34 studies published between 2000 and 2015 and found that the prevalence of SA ranged from 1.8% to 34% (Fedina et al., 2018). A global review of 35 studies on sexual aggression found that prevalence rates for victimization ranged from 0.4% to 85% (Hernández-Romero et al., 2019), but the authors did not disaggregate by gender identity. The range in prevalence estimates across these reviews could be contributed to methodological disparities in how SA is defined and measured. For example, across campus climate studies, there is discrepancy in the time in which experiences of SA are asked about, with some surveys asking about past year experiences and others asking about the entire time in HE. Further, different forms or types of SA may be prevalent, which must be accounted for when synthesizing estimates. In this study, we explore the vast discrepancy across prevalence estimates found in previous reviews by systematically reviewing and meta-analyzing evidence on the prevalence of SA experienced by HE students, stratifying by type of SA of recall period to provide more precise estimates (García-Moreno et al., 2013).

Synthesizing self-reported data on SA among HE students is essential for informing the design and evaluation of prevention and response interventions. Universities are increasingly developing procedures and guidance on sexual consent training and bystander awareness and creating and adapting policies to address SA among students (Anderson & Whiston, 2005; Degue et al., 2014; Jouriles et al., 2018). Identifying the prevalence of SA experienced by HE students is critical for developing and monitoring programs and policies tailored for different environments, as developing effective and sustainable interventions will require a comprehensive understanding of the problem starting with a nuanced picture of the prevalence of SA in HE contexts (Allan et al., 2006; Bacchi, 2000). It is also critical, as countries outside of the United States are beginning to implement similar types of surveys in HEs to measure SA (Bull et al., 2022) and could benefit from a synthesis of the strengths and limitations of the existing evidence base.

Therefore, in this study, we aimed to gather and synthesize the available evidence on the prevalence of SA at HEs through achieving the following objectives:

1. Establish an evidence base of studies that quantitatively measure the prevalence of SA in HE.
2. Formally assess characteristics of studies and identify geographical and demographic gaps in research.
3. Synthesize the prevalence estimates from these studies using meta-analyses.
4. Provide separate analyses based on gender identity, world region, recall period, and type of SA experienced to unpack the vast discrepancies in estimates from existing studies.

## Methods

This systematic review with meta-analyses followed guidance outlined in the *Cochrane Handbook for Systematic Reviews of Interventions* and followed PRISMA reporting guidance, adapting the recommendations when necessary to be appropriate for a systematic review of prevalence estimates (Higgins & Green, 2008). Prior to conducting the review, a protocol was written and published. A completed PRISMA checklist can be found in the Supplemental material.

### Inclusion and Exclusion Criteria

Included studies were required to meet the following criteria:

1. The sample population was students attending HE during the data collection period.
2. The outcome was self-reported SA (defined as any unwanted attempted or completed sexual contact) while a student was a registered student.
3. The data collection method was a cross-sectional quantitative survey (prospective studies were included if their first measurement time point assessed experiences of SA after entering HE) (Munn et al., 2015).

These inclusion criteria varied from the review protocol by including all cross-sectional survey designs regardless of whether they used probabilistic random sampling. There were no geographical limits placed on the location of included studies. The search was conducted in four languages: English, French, Italian, and Spanish. Studies published in any of these languages were included with the assistance of translators.

Studies were excluded if they fit any of the below criteria:

1. Reported on young adults but did not restrict the sample to HE students.
2. Measured sexual harassment and not SA.
3. Reported only on SA experiences of HE educators or administration/staff.
4. Only assessed lifetime SA prevalence or SA prevalence after the age of 14.
5. Were qualitative, reported on an intervention, or assessed vignettes, hypothetical situations, or attitudes about SA.

### Literature Search

The search terms were piloted and refined, considering both sensitivity and specificity (Higgins & Green, 2008) (see Table 1). In August 2020, this search strategy was implemented in Medline, EMBASE, Global Health, PsycINFO, Web of Science, ERIC, and CINAHL. Additionally, LILACS, a regional database, was used to search in English and Spanish. Further, Google Scholar used to search in French, Spanish, and Italian. We also searched relevant grey literature publications, including theses and dissertations. We conducted forward and backward citation tracking. The search was updated in May 2022. See Table 1 for an example of how the search strategy was executed.

**Table 1.** Example Search Strategy.

1.	(((sex* or intercourse) and (assault* or aggress* or harass* or violen* or abus* or nonconsensual or forced)) or rape*).ti. or exp rape/
2.	(college* or universit* or post?secondary or higher?education or undergraduate or graduate or campus*).tw. or exp universities/
3.	(survey* or prevalence or epidemiolog* or incidence or observational).tw. or *prevalence/
4.	1 and 2 and 3

### Screening, Coding Procedures, and Data Extraction

After completing the search, records were imported into the reference manager software Covidence, and duplicates were removed. BS first screened titles and abstracts of studies according to the set of pre-specified inclusion and exclusion criteria and then screened selected full-text studies. Following best practice guidance, a second reviewer (MM) also screened a random 10% of the articles at both stages (Lombard et al., 2002). Interrater reliability was 92%. Disagreements were resolved through consensus between the research team.

The following information was extracted from each article: the definition of SA used; the tool used to measure SA; the country where the study took place; the World Health Organization’s (WHO) global region where the country is situated (Popova et al., 2017) (African; Eastern Mediterranean; European; Americas; South-East Asia; Western Pacific); the sampling method; the study sample size; the response rate; the prevalence estimates of each type of SA (see Table 2); and the prevalence estimates disaggregated by gender identity. When studies reported on gender identities other than men and women, for example, transgender (Hoxmeier, 2016), gender minority (Parr, 2020), or genderqueer or nonconforming (Cantor et al., 2020; Cusano et al., 2023), these prevalence estimates were meta-analyzed together under the category transgender and gender-diverse people due to the small number of studies that reported on this subgroup. When studies did not report SA disaggregated by gender identity, prevalence estimates from these studies were extracted and labeled as gender aggregated estimates.

**Table 2.** Definitions for Types of Sexual Assault.

Type SA	Definition
Rape	Unwanted sexual penetration
Attempted rape	Unwanted attempted sexual penetration
Coercive sex	Unwanted sexual penetration or sexual touching that occurs when the perpetrator uses their position of authority, offers bribes in exchange for sexual activity, or applies nonphysical threats, such as ending the relationship
Forced sexual touching	Unwanted contact by force, threat, or incapacitation that is sexual in nature but does not involve sexual penetration

When studies reported a total estimate of SA or a single estimate of SA (i.e., rape), this estimate was included in the meta-analyses of the total prevalence. When studies presented estimates disaggregated by type of SA (see Table 2), multiple estimates were extracted. The data extraction was an iterative process, where estimates were eventually grouped together based on types of SA following a mapping process. To ensure reliability and replicability in the data extraction process, two reviewers extracted data for 10% of included studies. No discrepancies were identified in this process.

Prevalence estimates were recorded as the proportion of participants from the study sample who had experienced each type of SA. If reported, the proportions were extracted directly from a study. If not directly reported, the proportions were calculated by dividing the number of participants who experienced SA by the total number of participants (Munn et al., 2015). Proportions were stratified by gender identity, type of SA, and global region, where the data allowed. This subgroup analysis also was conducted to explore possible causes of heterogeneity among study results.

### Analysis

Meta-analyses were conducted to synthesize results of the included studies. The pooled prevalence estimates were determined using a random-effects generalized linear mixed model with logit transformed proportions (Munn et al., 2015). A total estimate of SA for each gender identity was assessed, and then, for each gender identity, the estimates were stratified by recall period and type of SA, as well as geographic location. Estimates from studies that did not disaggregate estimates by gender identity were pooled separately. When studies reported findings based on the same samples, the study that provided the most detailed information was included in the meta-analysis (Stoltenborgh et al., 2012). Each participant was only included once in each meta-analysis.

The pooled prevalence estimates were arrived at through meta-analyses that included studies that were based on any kind of sample (e.g., random, convenience, not specified). This differed from the PROSPERO protocol, which stated that the primary studies needed to use probabilistic random sampling. The intention of the protocol was to only include studies that employed rigorous methodologies (such as probabilistic random sampling) to reduce response bias from self-selection. However, given the emerging evidence that accurate prevalence rates on SA in campus climate surveys can be obtained through a variety of different sampling strategies (Jeffrey et al., 2022; Rosenthal & Freyd, 2018), we made the decision to not restrict the inclusion criteria to a specific sampling strategy. The implications of this decision are discussed in the risk of bias and discussion sections of this paper. When the same study was reported on by more than one article, one article for each study was selected. This selection was made by selecting the article that was most comprehensive and reported the most disaggregated results for the outcomes of interest in this review. Results were presented with 95% confidence intervals (95% CIs) (Higgins & Green, 2008). All meta-analyses were conducted in R Statistical Software (v4.2.1) using metafor (Viechtbauer, 2010) and lme4 (Bates et al., 2015) packages.

Heterogeneity between studies was assessed by the *I*^2^ index. To address the potential of high heterogeneity, meta-analyses were conducted using subgroups and as recommended by Migliavaca et al. (2022) and IntHout et al. (2016), 95% prediction intervals (95% PIs), in addition to CIs were reported for each meta-analyzed estimate. A 95% PI estimates the expected true prevalence estimate for 95% of future comparable studies (IntHout et al., 2016). Prediction intervals are useful for understanding the variability and uncertainty surrounding prevalence estimates when there is substantial variation in estimates across studies settings (IntHout et al., 2016). The implications of the heterogeneity were assessed and discussed in the discussion section of this article.

After all prevalence estimates were extracted, a review of the terms, definitions, different sexually violent acts, and categories of SA reported in the included studies was conducted and visualized. This process involved extracting the terms and definitions of each included study. Understanding how included studies define SA was essential for understanding how SA was defined, conceptualized, and measured in each included study.

### Quality of Studies: Risk of Bias Assessment

We assessed study risk of bias using The Joanna Briggs Institute’s Checklist for Prevalence Studies (2017). The following nine criteria were assessed:

1. Was the sample frame appropriate to address the target population?
2. Were study participants sampled in an appropriate way?
3. Was the sample size adequate?
4. Were the study subjects and the setting described in detail?
5. Was the data analysis conducted with sufficient coverage of the identified sample?
6. Were valid methods used for the identification of the condition?
7. Was the condition measured in a standard, reliable way for all participants?
8. Was there appropriate statistical analysis?
9. Was the response rate adequate, and if not, was the low response rate managed appropriately?

Each criterion was given a score of 1 if the criterion was met, 0 if the criterion was not met, and NA (scored as 0) if the criterion was not applicable.

## Results

### Overview of Included Studies

**Figure 1.**
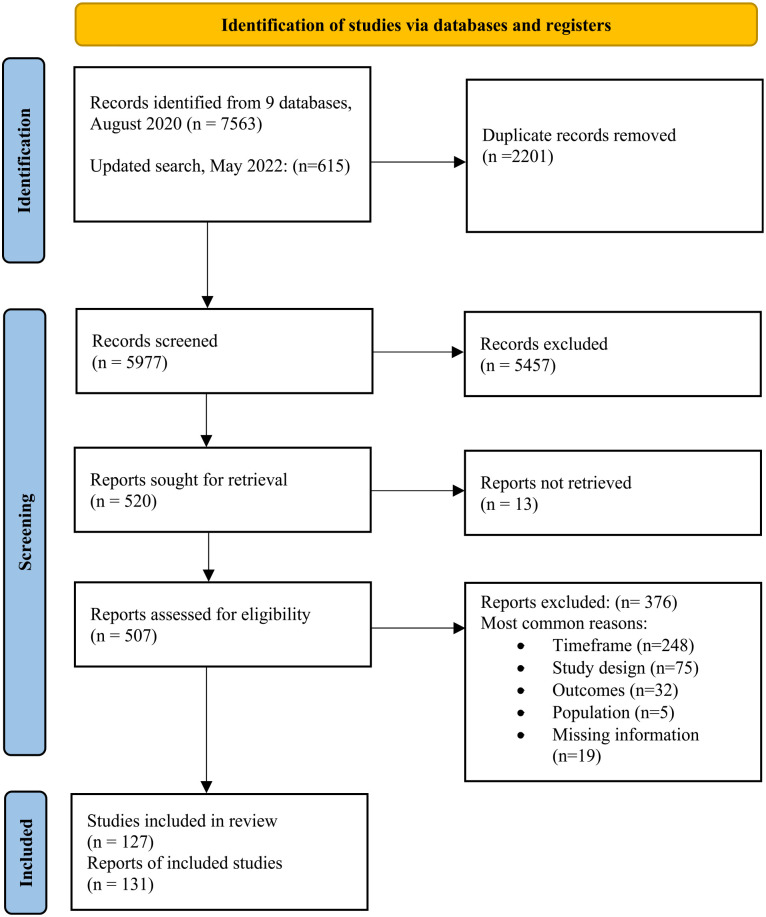
PRISMA flow diagram.

The search retrieved 5,977 articles. Of which, 131 articles were included with 113 reporting on women’s victimization, 55 reporting on men’s victimization, 11 reporting on victimization of transgender or gender-nonbinary people, and 43 reporting only gender aggregated victimization. The most common reason for excluding articles was that a study had a recall period for SA that predated their time in HE (e.g., Osman, 2016). The PRISMA flowchart (Figure 1) outlines the study selection process (Moher et al., 2009).

**Figure 2.**
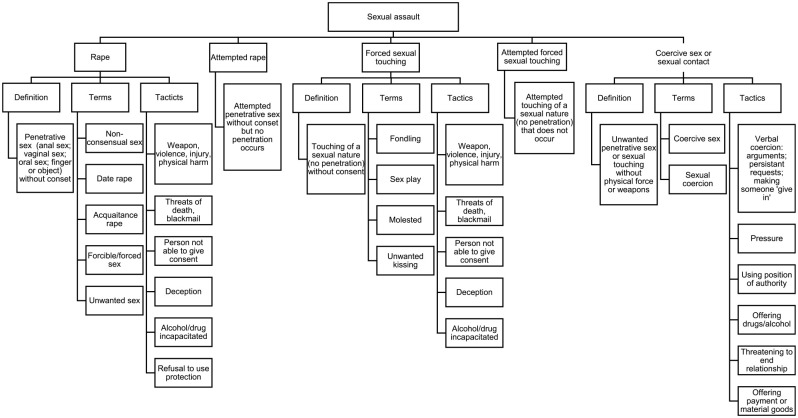
Defining sexual assault based on included studies.

Included articles were published from 1982 to 2022. The most commonly used SA measure was Koss’ Sexual Experiences Survey (SES) (*n* = 42 or 32% of articles). SA was defined differently across included studies. We identified four overarching types of SA reported on included studies: rape (unwanted sexual penetration by force, incapacitation, or threat), attempted rape (attempted unwanted sexual penetration by force, incapacitation, or threat), forced sexual touching (unwanted contact by force, threat, or incapacitation that is sexual in nature but does not involve sexual penetration), and coercive sex (unwanted penetration or sexual touching that occurs when the perpetrator uses their position of authority, offers bribes in exchange for sexual activity, or applies nonphysical threats, such as ending the relationship) (see Table 2). Figure 2 outlines how terms used in included studies were categorized into these types of SA and provides a detailed definition for each type of SA.

The samples in each article varied from *n* = 71 to *n* = 64, 000. Most articles (73%) used samples from the United States (*n* = 95) followed by Canada contributing samples to 5% of included articles (*n* = 7). Eleven countries contributed only one study. In terms of WHO regions, only one study was from the South-East Asian region, and no included studies were from the Eastern Mediterranean region. The recall periods measured in each article ranged from within the past year (*n* = 66) to throughout the entire time in HE (*n* = 69). Table 3 provides information on the characteristics of included articles.

**Table 3.** Summary of Study Characteristics.

Characteristics				
	Median (IQR)	Range	*N*	%
Sampling strategy				
Total population or random sampling			69	51.9
Convenience sampling			54	41.2
Did not report			10	6.9
Gender^a^				
Included men			55	41.2
Included women			111	84.0
Included transgender and gender-diverse people			11	8.4
Included gender aggregated			45	32.8
Recall period^b^				
Past year experiences			67	50.4
Entire time in higher education			70	52.7
Geographical setting				
African region			8	6.1
Region of the Americas			107	80.9
Eastern Mediterranean region			0	0
European region			10	7.6
South-East Asian region			1	0.8
Western Pacific region			7	4.6
Sample size	869 (321, 3,611)	102–64,005		
Response rate^c^	0.46 (0.25, 0.69)	0.05–0.98		

a Total exceeds 100% as some studies included both men and women.

b Total exceeds 100% as some studies asked about SA both in the past year and in the entire time in higher education.

c Sixty-nine articles did not report.

*Note*. SA = Sexual assault; IQR = Interquartile range.

### Risk of Bias

We identified some bias in included articles (see Supplemental material). The most common reason for increased risk of bias was the sampling strategy. While many studies used an appropriate sampling frame (total population, representative sample, or a random sample), convenience sampling was used in 54 studies. Convenience sampling can be problematic as it does not provide a representative sample of the population and can lead to nonresponse bias (when survey respondents have different characteristics than survey nonrespondents) (Lavallée & Beaumont, 2015). Specifically for surveys on SA in HE settings, women have been found to be more likely to respond than men (Giroux et al., 2020). Further, individuals who have experienced SA may be more or less likely to respond to surveys on SA; however, there is limited research in the field exploring this potential bias. Low response rates can also contribute to risk of bias, with 31 including studies reporting insufficient response rates. Finally, there was a lack of reporting on the tool used to measure SA; 44 studies did not report how SA was measured. These inconsistencies have the potential to limit the overall quality and interpretability of the pooled effect sizes.

### Prevalence

**Figure 3.**
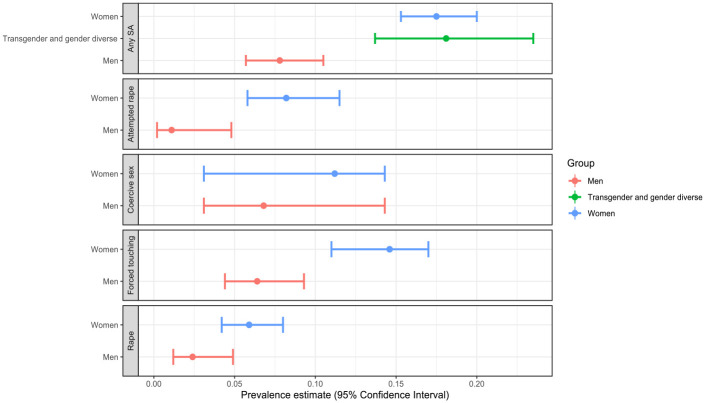
Sexual assault prevalence disaggregated by type.

Globally, for HE students, the prevalence of any type of SA victimization was 11% (95% CI [9.0, 13.3]; 95% PI [2.7, 35.1]). Prevalence varied by gender with 17.5% of women experiencing any type of SA ([15.3, 20.0]; [4.2, 52.4]), compared to 7.8% of men ([5.7, 10.5]; [0.9, 41.2]; see Table 4 and Figure 3). Transgender and gender-diverse students experienced 18.1% SA victimization ([13.7, 23.5]; [5.6, 40.0]). All meta-analyses had considerable heterogeneity (*I*^2^ > 97%).

**Table 4.** Meta-Analyzed Prevalence of SA Victimization Stratified by Recall Period and Type of SA.

Types of SA	Recall Period		
	Past Year Prevalence With Confidence Intervals	Entire Time at An HEI Prevalence With Confidence	Overall Prevalence With Confidence Intervals
Rape			
Women	0.051 [0.034, 0.076] studies: 29	0.07 [0.043, 0.111] studies: 29	0.059 [0.042, 0.080] studies: 58
Men	0.015 [0.007, 0.036] studies:12	0.049 [0.018, 0.126] studies: 9	0.024 [0.012, 0.049] studies: 21
Transgender or gender-diverse	NA	NA	NA
Gender aggregated	NA	NA	NA
Attempted rape			
Women	0.081 [0.051, 0.124] studies: 15	0.084 [0.048, 0.142] studies: 11	0.082 [0.058, 0.115] studies: 26
Men	0.005 [0.001, 0.022] studies: 7	0.07 [0.007, 0.449] studies: 3	0.011 [0.002, 0.048] studies: 10
Transgender or gender-diverse	NA	NA	NA
Gender aggregated	NA	NA	NA
Forced sexual touching			
Women	0.152 [0.108, 0.208] studies: 25	0.139 [0.095, 0.199] studies: 22	0.14 [0.11, 0.17] studies: 47
Men	0.077 [0.046, 0.126] studies: 14	0.053 [0.032, 0.089] studies: 7	0.064 [0.044, 0.093] studies: 21
Transgender or gender-diverse	NA	NA	NA
Gender aggregated	NA	NA	NA
Coercive sex			
Women	0.114 [0.065, 0.193] studies: 13	0.113 [0.063, 0.194] studies: 12	0.112 [0.031, 0.143] studies: 25
Men	0.062 [0.021, 0.172] studies: 6	0.079 [0.029, 0.195] studies: 3	0.068 [0.031, 0.143] studies: 9
Transgender or gender-diverse	NA	NA	NA
Gender aggregated	NA	NA	NA
Overall			
Women	0.180 [0.148, 0.216] studies: 47	0.168 [0.138, 0.203] studies: 50	0.175 [0.153, 0.20] studies: 97
Men	0.09 [0.060, 0.135] studies: 27	0.063 [0.040, 0.096] studies: 20	0.078 [0.057, 0.105] studies: 47
Transgender or gender-diverse	NA	NA	0.181 [0.137, 0.235] studies: 8
Gender aggregated	0.102 [0.068, 0.149] studies: 13	0.114 [0.086, 0.149] studies: 24	0.110 [0.087, 0.137] studies: 37

*Note*. NA indicates that meta-analyses were not possible due to the number of included studies. SA = Sexual assault; HE = higher education.

Separate meta-analyses for each type of SA by gender are shown in Table 4 and Figure 3. Across all types of SA, we found that women experienced higher prevalence rates when compared to men. Due to a small number of studies, we were not able to conduct separate analyses for types of SA experienced by transgender and gender diverse students. For both men and women students, forced sexual touching was the most common SA act, followed by coercive sex. Attempted rape and rape were experienced by a much smaller proportion of both men and women students.

**Figure 4.**
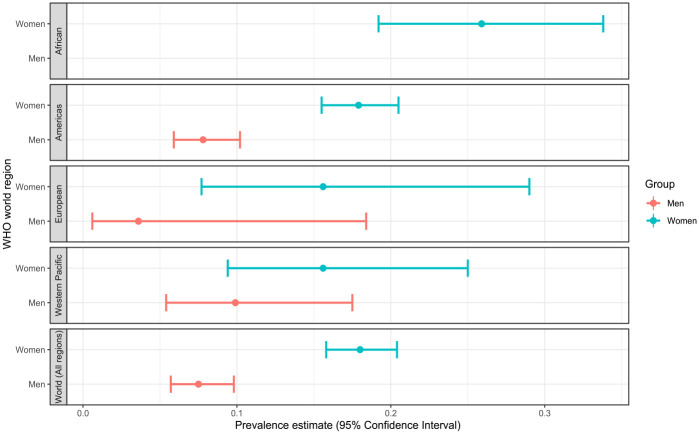
Sexual assault prevalence disaggregated by WHO world region.

The prevalence of SA also varied based on the world region where students attended HE (Figure 4 and Table 5). We found that the African Region had the highest prevalence of SA among women at 25.9% (95% CI [19.2, 33.8]; 95% PI [10.2, 51.7]), followed by the Americas at 17.9% ([15.5, 20.5]; [4.4, 50.5]), which was the region with the most studies (*n* = 86). The prevalence of any type of SA experienced by women in the European Region and the Western Pacific Region was only slightly lower than the prevalence found in the Americas (Table 5).

**Table 5.** Sexual Assault Prevalence Disaggregated by WHO World Region.

World Region	
	Overall Prevalence With Confidence Intervals
African region	
Women	0.259 [0.192, 0.338] studies: 8
Men	One study
Transgender or gender-diverse	No studies
Gender aggregated	One study
Region of the Americas	
Women	0.179 [0.155, 0.205] studies: 86
Men	0.078 [0.059, 0.102] studies: 43
Transgender or gender-diverse	0.181 [0.137, 0.235] studies: 8
Gender aggregated	0.112 [0.092, 0.135] studies: 37
South-East Asian region	
Women	One study
Men	No studies
Transgender or gender-diverse	No studies
Gender aggregated	No studies
European region	
Women	0.156 [0.077, 0.290] studies: 9
Men	0.036 [0.006, 0.184] studies: 5
Transgender or gender-diverse	No studies
Gender aggregated	0.063 [0.021, 0.173] studies: 4
Eastern Mediterranean region	
Women	No studies
Men	No studies
Transgender or gender-diverse	No studies
Gender aggregated	No studies
Western Pacific region	
Women	0.156 [0.094, 0.250] studies: 6
Men	0.099 [0.054, 0.175] studies: 5
Transgender or gender-diverse	No studies
Gender aggregated	0.144 [0.085, 0.234] studies: 5

Only three of six WHO world regions had estimates for men’s SA victimization, and the vast majority were from the Americas. The prevalence of victimization among men was more than double in the Americas, 7.8% (95% CI [5.9, 10.2]; 95% PI [1.2, 36.3]), compared to the European region. However, the Western Pacific Region had the highest rates of men’s SA at 9.9% (95% CI [5.4, 17.5]; 95% CI [2.2, 35.0]) (Table 5). An overview of the critical findings from the review can be found in Table 6.

**Table 6.** Critical Findings.

• Our search identified 131 articles from 21 different countries reporting on the prevalence of SA among HE students. • Through meta-analyses we found that 17.5% of women, 7.8% men, and 18.1% of transgender and gender diverse people experienced SA as HE students. • Forced sexual touching was the most common act of SA experienced by HE students when compared to attempted and completed rape and coerced sex. • While most of the research was from the U.S. context, we found that the African Region had the highest prevalence estimates for women’s SA and the Western Pacific region had the highest prevalence estimates for men’s SA. • There was considerable heterogeneity across included studies, even when estimates of SA were disaggregated by type of SA, gender identity, and world region.

*Note*. HE = higher education; SA = Sexual assault.

## Discussion

Across all included studies, we found that 17.5% of women and 7.8% of men HE students have experienced SA. We found that women faced higher rates of all types of SA (rape, attempted rape, coerced sex, and forced sexual touching) when compared to men, which has repeatedly been found within existing research on SA in HE settings in the United States context (Cantor et al., 2019, 2020; Fedina et al., 2018). While only a small number of included studies reported on SA among transgender and gender-diverse samples, we found that this population experienced the highest rates of SA (18.1%). This finding is consistent with research outside of the HE context (Stotzer, 2009) and should be used to inform prevention and responses initiative in HE settings that often unintentionally exclude transgender and gender-diverse communities (Gretgrix & Farmer, 2023).

The most common act of SA reported in included studies was forced sexual touching, irrespective of gender identity. This act was more common than coercive sex, attempted rape, or rape. This finding aligns with the review on SA prevalence at U.S. HEs conducted by Fedina et al. (2018), which found consistently higher rates of unwanted sexual contact across included studies, when compared to rape. Disaggregating between different types of meta-analyses is a key strength to our research as it helps to address the vast range of estimates found in previous reviews. Understanding the types of SA that are most common among HE students is also important for designing tailored and effective prevention initiatives and response services that reflect student experiences.

At the global level, although there remains limited evidence from the South-East Asian and Eastern Mediterranean region, especially for men, the meta-analyzed estimates showed that women reported consistently higher rates of SA victimizations in each region and were most at risk in the African Region. This finding makes an important contribution to the body of evidence on SA at HEs. A meta-analysis of the worldwide prevalence of SA (Abrahams et al., 2014) found that in 2010, 7.2% of women had experienced nonpartner SA, with the highest prevalence rates in Sub-Saharan Africa. We similarly found that across included studies measuring SA in HE the prevalence of SA was highest for women in studies from the African Region. However, we also found that all meta-analyzed regional estimates on SA prevalence among HE students were more than double than what Abrahams et al. (2014) found in the general population, indicating that HE could be a particularly risky place for experiencing SA. It might not seem intuitive that HE student could have higher prevalence rates of SA when compared to the general population. This environment is populated by highly educated people who are presumably aware of their rights and responsibilities. Yet, SA most often occurs between the ages of 18 and 24 years (Basile et al., 2007), most survivors know the perpetrator (Avegno et al., 2009; Planty et al., 2013), and incidences are often linked to environments where there is alcohol (Abbey et al., 2001), absence of guardianship or effective bystanders (Gialopsos, 2017), and men’s peer support for SA (Yapp & Quayle, 2018). As a result, HEIs, where many of these risk factors converge, could be particularly risking settings for SA.

This paper further contributes to the literature on SA in HE institutions by meta-analyzing men’s experiences with SA. While significantly less prevalent when compared to women and gender-diverse samples, we found that a substantial proportion of men have experienced SA in HE (7.8%). This finding contradicts traditional notions of men’s SA as “invisible and incomprehensible” (Hlavka, 2017, p. 482). Men’s experiences of SA may uniquely involve certain stigmas around shame, embarrassment, and disbelief, centered around societal constructions of masculinity, dominance, and power (Hlavka, 2017). The way in which men experience SA and the stigmas men face around reporting SA can also be different than women, which has theoretical and methodological implications for the field of SA in HE research (Hlavka, 2017). However, like women and transgender and gender-diverse people, men’s experiences of SA cannot be understood uniformly. Men who identify as gay and bisexual have been found to experience SA at higher rates than heterosexual men (Bullock & Beckson, 2011; Donne et al., 2018). Future analyses could be conducted to understand men’s SA in a more nuanced way and to further contribute to the limited existing evidence base on men’s SA in HEIs. Additionally, more research is needed on men’s SA, especially outside of the Americas. In this study, regional estimates for men were not possible due to a lack of studies in three of the six WHO regions, including the African Region, which reported the highest rates of SA among women. The Western Pacific Region had the highest estimates for men’s SA, however there were only five studies included in the analysis.

The SES, a valid and reliable tool o measure SA, was the most commonly used tool among included studies (Koss & Gidycz, 1985). One of the key strengths of this measure is its use of behaviorally specific language to measure SA instead of asking explicitly about “rape” or “assault” (Canan et al., 2020). In doing so, the tool is able to capture the experiences of participants who may not see themselves as “victims” or who may not identify their experience as assault. Included studies that did not use valid or reliable measures may produce different, and lower, prevalence estimates. A limitation of this review is that separate meta-analyses were not conducted for each type of SA measure used. This is something that could be explored in future research.

There are several limitations to this review. Although this is the first systematic review to examine the prevalence of SA at HEs outside of the United States, the searchers were still limited to only four languages (English, French, Spanish, and Italian). There may be opportunities in future research to expand the remit of searches to other global regions not covered here. Second, all meta-analyses had considerable heterogeneity (*I*^2^ > 97%), which potentially limits the reliability and the generalizability of the pooled estimates. Despite the presence of heterogeneity in study characteristics, synthesizing the evidence was important given the abundance of studies at the local level that had not been captured by U.S.-focused reviews (Munn et al., 2015). This is important, as included studies from outside the United States report high rates of SA. For example, the one included study from Turkey found that 45.2% of women and 39.6% of men have experienced SA in the past year (Schuster et al., 2016), and the one included study from Eswatini found that 38% of women experienced SA and 20% experienced rape in the past year (Fielding-Miller et al., 2019). Another limitation to the evidence base is the potential presence of nonresponse bias in included studies (where survey respondents have different characteristics than survey nonrespondents) (Brunton-Smith et al., 2022). For example, the propensity to respond to a survey might be different for people based on their exposure to SA (Brunton-Smith et al., 2022). Nonresponse bias is a particular concern in included studies that used convenience sampling and studies with low response rates; declining survey participation rates due to student survey fatigue and lack of institutional trust has been deemed a threat to the efficacy of SA surveys in HEs (Tschepikow, 2012). Further research is needed to understand and address the direction and magnitude of nonresponse bias in research on SA in HE (Fosnacht et al., 2017).

Finally, the SES, a valid and reliable tool to measure SA, was the most frequently used tool among included studies (Koss & Gidycz, 1985). One of the key strengths of this measure is its use of behaviorally specific language to measure SA instead of asking explicitly about “rape” or “assault” (Canan et al., 2020). In doing so, the tool can capture the experiences of participants who may not see themselves as “victims” or who may not identify their experience as assault. Included studies that did not use valid or reliable measures may produce different, and lower, prevalence estimates. A limitation of this review is that separate meta-analyses were not conducted for each type of SA measure used. This is something that could be explored in future research.

Quantifying the prevalence and burden of SA experienced by students has implications for policy and programming change. The findings (summarized in Table 6) identify the potential scale of SA among HE students globally and demonstrate the need for intervention to prevent and respond to SA among this population. The findings also point to the need to have standardized methods and tools for measuring and reporting on SA in HE to produce more valid and reliable estimates from campus climate surveys and address the vast range of estimates found across the literature (see Table 7). HE can have transformative outcomes throughout the life course (Mayhew et al., 2016), but the results of this research suggest that safety concerns exist for students pursuing this education across the world. With an increasing amount of research highlighting the scope and consequences of SA globally, the HE sector is and will continue to face increasing pressure to protect the safety and well-being of students. Ultimately, universities and policy makers may be more inclined to dedicate financial resources and capacity to preventing and responding to incidences of SA if there is an estimate of the scope and pervasiveness of the problem.

**Table 7.** Implications for Practice, Policy, and Research.

Practice	• HE institutions, especially those outside of the United States, should commit to the implementation of campus climate surveys to monitor SA prevalence. • HE institutions should dedicate increased resources to supporting student survivors of SA.
Policy	• Governments and policy makers, globally, should prioritize, resource, and mandate SA prevention and response interventions for HE students.
Research	• To address the vast range of prevalence estimates found across the literature, there is a need for more standardized methods and tools for measuring and reporting on SA in HE that produce more valid and reliable estimates from campus climate surveys. • More consistent and coordinated research is needed on SA on HE students outside of the United States context.

*Note*. HE = higher education; SA = Sexual assault.

## Supplemental Material

sj-docx-1-tva-10.1177_15248380231196119 – Supplemental material for The Prevalence of Sexual Assault Among Higher Education Students: A Systematic Review With Meta-Analyses Supplemental material, sj-docx-1-tva-10.1177_15248380231196119 for The Prevalence of Sexual Assault Among Higher Education Students: A Systematic Review With Meta-Analyses by Bridget Steele, Mackenzie Martin, Alessandra Sciarra, G. J. Melendez-Torres, Michelle Degli Esposti and David K. Humphreys in Trauma, Violence, & Abuse

sj-docx-2-tva-10.1177_15248380231196119 – Supplemental material for The Prevalence of Sexual Assault Among Higher Education Students: A Systematic Review With Meta-Analyses Supplemental material, sj-docx-2-tva-10.1177_15248380231196119 for The Prevalence of Sexual Assault Among Higher Education Students: A Systematic Review With Meta-Analyses by Bridget Steele, Mackenzie Martin, Alessandra Sciarra, G. J. Melendez-Torres, Michelle Degli Esposti and David K. Humphreys in Trauma, Violence, & Abuse

sj-docx-3-tva-10.1177_15248380231196119 – Supplemental material for The Prevalence of Sexual Assault Among Higher Education Students: A Systematic Review With Meta-Analyses Supplemental material, sj-docx-3-tva-10.1177_15248380231196119 for The Prevalence of Sexual Assault Among Higher Education Students: A Systematic Review With Meta-Analyses by Bridget Steele, Mackenzie Martin, Alessandra Sciarra, G. J. Melendez-Torres, Michelle Degli Esposti and David K. Humphreys in Trauma, Violence, & Abuse

sj-docx-4-tva-10.1177_15248380231196119 – Supplemental material for The Prevalence of Sexual Assault Among Higher Education Students: A Systematic Review With Meta-Analyses Supplemental material, sj-docx-4-tva-10.1177_15248380231196119 for The Prevalence of Sexual Assault Among Higher Education Students: A Systematic Review With Meta-Analyses by Bridget Steele, Mackenzie Martin, Alessandra Sciarra, G. J. Melendez-Torres, Michelle Degli Esposti and David K. Humphreys in Trauma, Violence, & Abuse

sj-docx-5-tva-10.1177_15248380231196119 – Supplemental material for The Prevalence of Sexual Assault Among Higher Education Students: A Systematic Review With Meta-Analyses Supplemental material, sj-docx-5-tva-10.1177_15248380231196119 for The Prevalence of Sexual Assault Among Higher Education Students: A Systematic Review With Meta-Analyses by Bridget Steele, Mackenzie Martin, Alessandra Sciarra, G. J. Melendez-Torres, Michelle Degli Esposti and David K. Humphreys in Trauma, Violence, & Abuse

sj-docx-6-tva-10.1177_15248380231196119 – Supplemental material for The Prevalence of Sexual Assault Among Higher Education Students: A Systematic Review With Meta-Analyses Supplemental material, sj-docx-6-tva-10.1177_15248380231196119 for The Prevalence of Sexual Assault Among Higher Education Students: A Systematic Review With Meta-Analyses by Bridget Steele, Mackenzie Martin, Alessandra Sciarra, G. J. Melendez-Torres, Michelle Degli Esposti and David K. Humphreys in Trauma, Violence, & Abuse
