# Supplementary material for: The Prevalence of Sexual Assault Among Higher Education Students: A Systematic Review With Meta-Analyses
Source: Trauma Violence Abuse. 2023 Sep 20;25(3):1885–98. doi: 10.1177/15248380231196119 (PMC11155219; doi:10.1177/15248380231196119)
Supplement: sj-docx-2-tva-10.1177_15248380231196119 – Supplemental material for The Prevalence of Sexual Assault Among Higher Education Students: A Systematic Review With Meta-Analyses [file sj-docx-2-tva-10.1177_15248380231196119.docx]

Full search strategy for one database

**Medline (Ovid MEDLINE® Epub Ahead of Print, In-Process & Other Non-Indexed Citations, Ovid MEDLINE® Daily and Ovid MEDLINE®) 1946 to present**

| 1. | (((sex* or intercourse) and (assault* or aggress* or harass* or violen* or abus* or non-consensual or forced)) or rape*).ti. or exp rape/ |
| --- | --- |
| 2. | (college* or universit* or post?secondary or higher?education or undergraduate or graduate or campus*).tw. or exp universities/ |
| 3. | (survey* or prevalence or epidemiolog* or incidence or observational).tw. or *prevalence/ |
| 4. | 1 and 2 and 3 |
