# Supplementary material for: The Prevalence of Sexual Assault Among Higher Education Students: A Systematic Review With Meta-Analyses
Source: Trauma Violence Abuse. 2023 Sep 20;25(3):1885–98. doi: 10.1177/15248380231196119 (PMC11155219; doi:10.1177/15248380231196119)
Supplement: sj-docx-3-tva-10.1177_15248380231196119 – Supplemental material for The Prevalence of Sexual Assault Among Higher Education Students: A Systematic Review With Meta-Analyses [file sj-docx-3-tva-10.1177_15248380231196119.docx]

References for included studies:

1. Adejimi AA, Sabageh OA, Adedokun OP. Experiences and disclosures of sexual assault among Nigerian undergraduates in a tertiary institution. *Violence and gender* 2016; **3**(4): 208-15.

2. Adhia A, Ellyson AM, Kroshus E. Prevalence and formal reporting of sexual violence among undergraduate student-athletes: a multi-state study. *Journal of interpersonal violence* 2022: 08862605221081936.

3. Adinew YM, Hagos MA. Sexual violence against female university students in Ethiopia. *BMC international health and human rights* 2017; **17**(1): 1-7.

4. Ajayi AI, Mudefi E, Owolabi EO. Prevalence and correlates of sexual violence among adolescent girls and young women: findings from a cross-sectional study in a South African university. *BMC women's health* 2021; **21**(1): 1-9.

5. Ameral V, Palm Reed KM, Hines DA. An analysis of help-seeking patterns among college student victims of sexual assault, dating violence, and stalking. *Journal of Interpersonal Violence* 2020; **35**(23-24): 5311-35.

6. Amos C, Peters RJ, Williams L, Johnson RJ, Martin Q, Yacoubian GS. The link between recent sexual abuse and drug use among African American male college students: It's not just a female problem in and around campus. *Journal of psychoactive drugs* 2008; **40**(2): 161-6.

7. Andar S. The prevalence of college sexual assault among women in the digital media era: An online investigation of potential impacts from social networking, pornography, cyberbullying and sexting: Teachers College, Columbia University; 2014.

8. Anderson RE, Cahill SP, Delahanty DL. The psychometric properties of the Sexual Experiences Survey–Short Form Victimization (SES-SFV) and characteristics of sexual victimization experiences in college men. *Psychology of Men & Masculinity* 2018; **19**(1): 25.

9. Anthony ER, Cook SL. Assessing the impact of gender-neutral language on disclosure of sexual violence. *Psychology of Violence* 2012; **2**(3): 297.

10. Atkinson K. " An Amplified Space": A feminist poststructuralist analysis of sexual violence at university: Liverpool John Moores University (United Kingdom); 2020.

11. Backhaus I, Lipson SK, Fisher LB, Kawachi I, Pedrelli P. Sexual assault, sense of belonging, depression and suicidality among LGBQ and heterosexual college students. *Journal of American college health* 2021; **69**(4): 404-12.

12. Banyard VL, Demers JM, Cohn ES, et al. Academic correlates of unwanted sexual contact, intercourse, stalking, and intimate partner violence: An understudied but important consequence for college students. *Journal of interpersonal violence* 2020; **35**(21-22): 4375-92.

13. Banyard VL, Ward S, Cohn ES, Plante EG, Moorhead C, Walsh W. Unwanted sexual contact on campus: A comparison of women’s and men’s experiences. *Violence and victims* 2007; **22**(1): 52-70.

14. Barrick K, Krebs CP, Lindquist CH, Moore C, Plummer D. Factors associated with incidents of sexual assault among undergraduate women at historically black colleges and universities. *Victims & Offenders* 2012; **7**(2): 185-207.

15. Bell SC. The Relationship Between The Effects Of Rape, Posttraumatic Stress Disorder, Complex Trauma, And Post-Attack Victim-Perpetrator Interaction In Female College Students. 2015.

16. Beres MA, Stojanov Z, Graham K, Treharne GJ. Sexual assault experiences of university students and disclosure to health professionals and others. *New Zealand medical journal* 2020; **133**(1523): 55-64.

17. Bergeron M, Hébert M, Ricci S, et al. Violences sexuelles en milieu universitaire au Québec: Rapport de recherche de l’enquête ESSIMU. 2016.

18. Bird ER, Gilmore AK, George WH, Lewis MA. The role of social drinking factors in the relationship between incapacitated sexual assault and drinking before sexual activity. *Addictive behaviors* 2016; **52**: 28-33.

19. Blanco V, López L, Otero P, Torres ÁJ, Ferraces MJ, Vázquez FL. Sexual victimization and mental health in female university students. *Journal of interpersonal violence* 2022; **37**(15-16): NP14215-NP38.

20. Bryant NL. Child sexual abuse and its relationship to perceived vulnerability, powerlessness, self-efficacy, and sexual assault: Ohio University; 2000.

21. Cantor D, Fisher B, Chibnall SH, et al. Report on the AAU campus climate survey on sexual assault and sexual misconduct. 2015.

22. Cass AI. Routine activities and sexual assault: An analysis of individual-and school-level factors. *Violence and victims* 2007; **22**(3): 350-66.

23. Castaño-Castrillón JJ, González EK, Guzmán JA, et al. Acoso sexual en la comunidad estudiantil de la Universidad de Manizales (Colombia) 2008: estudio de corte transversal. *Revista Colombiana de Obstetricia y Ginecología* 2010; **61**(1): 18-27.

24. Chen L-R, Wang G-F, Xie G-D, et al. Association between sexual abuse victimization during the life course and suicidal behaviors in male and female college students in China: Timing, duration, types and patterns. *Journal of Affective Disorders* 2021; **280**: 30-8.

25. Chen R. Risk factors of sexual abuse among college students in Taiwan. *Journal of Interpersonal Violence* 1996; **11**(1): 79-93.

26. Choi EPH, Wong JYH, Fong DYT. An emerging risk factor of sexual abuse: the use of smartphone dating applications. *Sexual Abuse* 2018; **30**(4): 343-66.

27. Copenhaver S, Grauerholz E. Sexual victimization among sorority women: Exploring the link between sexual violence and institutional practices. *Sex Roles* 1991; **24**(1): 31-41.

28. Coulter RW, Mair C, Miller E, Blosnich JR, Matthews DD, McCauley HL. Prevalence of past-year sexual assault victimization among undergraduate students: Exploring differences by and intersections of gender identity, sexual identity, and race/ethnicity. *Prevention Science* 2017; **18**(6): 726-36.

29. Coulter RW, Rankin SR. College sexual assault and campus climate for sexual-and gender-minority undergraduate students. *Journal of interpersonal violence* 2020; **35**(5-6): 1351-66.

30. Curtis JK. Sexual assault on a college campus: The influences of alcohol consumption, alcohol expectancies, risky behaviors, and sorority membership. 2011.

31. Cusano J, Kirkner A, Johnson L, McMahon S. Sexual violence prevalence and disclosure patterns among college undergraduates: exploring types of sexual violence and incident-specific characteristics. *Journal of American college health* 2021: 1-11.

32. Daigle LE, Fisher BS, Cullen FT. The violent and sexual victimization of college women: Is repeat victimization a problem? *Journal of interpersonal violence* 2008; **23**(9): 1296-313.

33. Daigle LE, Johnston T, Azimi A, Felix SN. Violent and sexual victimization among American and Canadian college students: Who is more at risk and are the risk factors invariant? *Journal of School Violence* 2019; **18**(2): 226-40.

34. Dasgupta SM, A. Survey of 500 Women Finds 1 in 10 Had Been Sexually Assaulted in Higher Education Institutions. The WIRE. 2020.

35. DeKeseredy WS, Schwartz MD, Nolan J, Mastron N, Hall-Sanchez A. Polyvictimization and the continuum of sexual abuse at a college campus: does negative peer support increase the likelihood of multiple victimizations? *The British Journal of Criminology* 2019; **59**(2): 276-95.

36. DeKeseredy WS, Schwartz MD, Tait K. Sexual assault and stranger aggression on a candian university campus. *Sex Roles* 1993; **28**(5): 263-77.

37. Dion J, Boisvert S, Paquette G, Bergeron M, Hébert M, Daigneault I. Sexual violence at university: are indigenous students more at risk? *Journal of interpersonal violence* 2021: 08862605211021990.

38. Echeverría RE, Guerrero LP, Chim MDK, Cutz JLB, Trujillo CDC. Acoso y hostigamiento sexual en estudiantes universitarios: un acercamiento cuantitativo. *Enseñanza e investigación en psicología* 2017; **22**(1): 15-26.

39. Edwards KM, Sylaska KM, Barry JE, et al. Physical dating violence, sexual violence, and unwanted pursuit victimization: A comparison of incidence rates among sexual-minority and heterosexual college students. *Journal of interpersonal violence* 2015; **30**(4): 580-600.

40. Eisenberg ME, Lust K, Mathiason MA, Porta CM. Sexual assault, sexual orientation, and reporting among college students. *Journal of Interpersonal Violence* 2021; **36**(1-2): 62-82.

41. Fielding-Miller R, Shabalala F, Masuku S, Raj A. Epidemiology of campus sexual assault among university women in Eswatini. *Journal of interpersonal violence* 2021; **36**(21-22): NP11238-NP63.

42. Finkelson L, Oswalt R. College date rape: Incidence and reporting. *Psychological Reports* 1995; **77**(2): 526-.

43. Finley C, Corty E. Rape on the campus: The prevalence of sexual assault while enrolled in college. *Journal of College Student Development* 1993; **34**: 113-.

44. Fisher B, Cullen FT, Turner MG. The extent and nature of the sexual victimization of college women: A National Level Analysis: National Institute of Justice Washington, DC; 1999.

45. Fisher BS, Daigle LE, Cullen FT, Turner MG. Reporting sexual victimization to the police and others: Results from a national-level study of college women. *Criminal justice and behavior* 2003; **30**(1): 6-38.

46. Flack Jr WF, Daubman KA, Caron ML, et al. Risk factors and consequences of unwanted sex among university students: Hooking up, alcohol, and stress response. *Journal of Interpersonal Violence* 2007; **22**(2): 139-57.

47. Flack Jr WF, Hansen BE, Hopper AB, et al. Some types of hookups may be riskier than others for campus sexual assault. *Psychological trauma: theory, research, practice, and policy* 2016; **8**(4): 413.

48. Flack Jr WF, Kimble MO, Campbell BE, Hopper AB, Petercă O, Heller EJ. Sexual assault victimization among female undergraduates during study abroad: A single campus survey study. *Journal of interpersonal violence* 2015; **30**(20): 3453-66.

49. Ford J, Soto-Marquez JG. Sexual assault victimization among straight, gay/lesbian, and bisexual college students. *Violence and gender* 2016; **3**(2): 107-15.

50. Forke CM, Myers RK, Catallozzi M, Schwarz DF. Relationship violence among female and male college undergraduate students. *Archives of pediatrics & adolescent medicine* 2008; **162**(7): 634-41.

51. Fuentes-Pumarola C, Reyes-Amargant Z, Berenguer-Simon A, et al. Alcohol use and sexual violence among nursing students in Catalonia, Spain: a multicentre cross-sectional study. *International journal of environmental research and public health* 2021; **18**(11): 6036.

52. Gartner RE. From gender microaggressions to sexual assault: Measure development and preliminary trends among undergraduate women: University of California, Berkeley; 2019.

53. Gross AM, Winslett A, Roberts M, Gohm CL. An examination of sexual violence against college women. *Violence against women* 2006; **12**(3): 288-300.

54. Haughey E, Liu H, Lowenstein M, et al. Investigating the prevalence of non-consensual sexual experiences in a Northern Ireland university student population. HIV MEDICINE; 2018: WILEY 111 RIVER ST, HOBOKEN 07030-5774, NJ USA; 2018. p. S15-S.

55. Herbenick D, Fu T-C, Dodge B, Fortenberry JD. The alcohol contexts of consent, wanted sex, sexual pleasure, and sexual assault: Results from a probability survey of undergraduate students. *Journal of American college health* 2019; **67**(2): 144-52.

56. Herres J, Wang SB, Bobchin K, Draper J. A socioecological model of risk associated with campus sexual assault in a representative sample of liberal arts college students. *Journal of interpersonal violence* 2021; **36**(7-8): NP4208-NP29.

57. Hines DA, Armstrong JL, Reed KP, Cameron AY. Gender differences in sexual assault victimization among college students. *Violence and victims* 2012; **27**(6): 922-40.

58. Holland KJ. Correlates of college women’s intentions to use formal campus supports for sexual assault. *Psychology of violence* 2020; **10**(2): 245.

59. Holloway K, Bennett T. Alcohol-related rape among university students. *Victims & Offenders* 2018; **13**(4): 471-86.

60. Hossain MB, Memiah P, Adeyinka A. Are female college students who are diagnosed with depression at greater risk of experiencing sexual violence on college campus? *Journal of health care for the poor and underserved* 2014; **25**(3): 1341-59.

61. Howard DE, Griffin MA, Boekeloo BO. Prevalence and psychosocial correlates of alcohol-related sexual assault among university students. *Adolescence* 2008; **43**(172): 733.

62. Hoxmeier JC. Sexual assault and relationship abuse victimization of transgender undergraduate students in a national sample. *Violence and gender* 2016; **3**(4): 202-7.

63. Iliyasu Z, Abubakar IS, Aliyu MH, Galadanci HS, Salihu HM. Prevalence and correlates of gender-based violence among female university students in Northern Nigeria. *African journal of reproductive health* 2011; **15**(3): 123-33.

64. Johns TL. Alcohol and sexual aggression on campus: University of Florida; 2001.

65. Johnson LM, Matthews TL, Napper SL. Sexual orientation and sexual assault victimization among US college students. *The Social Science Journal* 2016; **53**(2): 174-83.

66. Jordan-Simmons KM. Sexual violence on campus: The relationship between sorority membership, fraternity contact, and alcohol consumption: University of Pittsburgh; 2001.

67. Jouriles EN, Nguyen J, Krauss A, Stokes SL, McDonald R. Prevalence of sexual victimization among female and male college students: a methodological note with data. *Journal of interpersonal violence* 2022; **37**(11-12): NP8767-NP92.

68. Kammer-Kerwick M, Wang A, McClain TS, et al. Sexual violence among gender and sexual minority college students: The risk and extent of victimization and related health and educational outcomes. *Journal of interpersonal violence* 2021; **36**(21-22): 10499-526.

69. Kilpatrick DG, Resnick HS, Ruggiero KJ, Conoscenti LM, McCauley J. Drug-facilitated, incapacitated, and forcible rape: A national study: National Criminal Justice Reference Service Charleston, SC; 2007.

70. Kimble M, Neacsiu AD, Flack WF, Horner J. Risk of unwanted sex for college women: Evidence for a red zone. *Journal of American College Health* 2008; **57**(3): 331-8.

71. Kirkner A, Plummer S-B, Findley PA, McMahon S. Campus sexual violence victims with disabilities: disclosure and help seeking. *Journal of interpersonal violence* 2022; **37**(9-10): NP7156-NP77.

72. Krebs CP, Barrick K, Lindquist CH, Crosby CM, Boyd C, Bogan Y. The sexual assault of undergraduate women at Historically Black Colleges and Universities (HBCUs). *Journal of interpersonal violence* 2011; **26**(18): 3640-66.

73. Krebs CP, Lindquist CH, Warner TD, Fisher BS, Martin SL. College women's experiences with physically forced, alcohol-or other drug-enabled, and drug-facilitated sexual assault before and since entering college. *Journal of American college health* 2009; **57**(6): 639-49.

74. Kullima AA, Kawuwa MB, Audu BM, Mairiga AG, Bukar M. Sexual assault against female Nigerian students. *African journal of reproductive health* 2010; **14**(3): 189-93.

75. Leone JM, Carroll JM. Victimization and suicidality among female college students. *Journal of American College Health* 2016; **64**(6): 421-8.

76. Lindquist CH, Barrick K, Krebs C, Crosby CM, Lockard AJ, Sanders-Phillips K. The context and consequences of sexual assault among undergraduate women at historically Black colleges and universities (HBCUs). *Journal of interpersonal violence* 2013; **28**(12): 2437-61.

77. Lott B, Reilly ME, Howard DR. Sexual assault and harassment: A campus community case study. *Signs: Journal of Women in Culture and Society* 1982; **8**(2): 296-319.

78. Luetke M, Giroux S, Herbenick D, Ludema C, Rosenberg M. High prevalence of sexual assault victimization experiences among university fraternity men. *Journal of interpersonal violence* 2021; **36**(23-24): 11755-67.

79. Lydston EE. Gender differences in sexual violence victimization and acknowledgement on a college campus: San Diego State University; 2016.

80. Magrin JV, Franco A, Makeeva I, Paranhos LR, Rigo L. Emotional, physical and sexual violence against female students undergoing medical, dental and psychology courses in South Brazil. *European journal of dental education* 2019; **23**(4): 455-60.

81. Maletsky LD. Gender Role Strain, Hookup Culture, and Sexual Violence: University of Nevada, Reno; 2019.

82. Marcantonio TL, Willis M, Schisler ED. Associations of alcohol consumption, sexual assault history, severity, and revictimization with college women’s bystander behaviors in alcohol-involved settings. *Journal of interpersonal violence* 2021; **36**(23-24): 11792-807.

83. Marsil DF, McNamara C. An examination of the disparity between self-identified versus legally identified rape victimization: A pilot study. *Journal of American college health* 2016; **64**(5): 416-20.

84. Martin Q, Peters Jr RJ, Amos Jr CE, et al. The relationship between sexual abuse and drug use: a view of African-American college students in Texas. *Journal of Ethnicity in Substance Abuse* 2005; **4**(1): 23-33.

85. McDougall EE, Langille DB, Steenbeek AA, Asbridge M, Andreou P. The relationship between non-consensual sex and risk of depression in female undergraduates at universities in maritime Canada. *Journal of interpersonal violence* 2019; **34**(21-22): 4597-619.

86. McMahon S, O’Connor J, Seabrook R. Not just an undergraduate issue: Campus climate and sexual violence among graduate students. *Journal of interpersonal violence* 2021; **36**(7-8): NP4296-NP314.

87. Mellins CA, Walsh K, Sarvet AL, et al. Sexual assault incidents among college undergraduates: Prevalence and factors associated with risk. *PLoS one* 2017; **12**(11): e0186471.

88. Mellins CA, Walsh K, Sarvet AL, et al. Sexual assault among undergraduates: Prevalence, ethnography, and recommendations for multilevel prevention. 65th Annual Meeting; 2018: AACAP; 2018.

89. Mennicke A, Coates CA, Jules B, Langhinrichsen-Rohling J. Who do they tell? College students’ formal and informal disclosure of sexual violence, sexual harassment, stalking, and dating violence by gender, sexual identity, and race. *Journal of interpersonal violence* 2021: 08862605211050107.

90. Mezie-Okoye M-MI, Alamina F. Sexual Violence among Female Undergraduates in a Tertiary Institution in Port Harcourt: Prevalence, Pattern, Determinants and Health Consequences. *African journal of reproductive health* 2014; **18**(4): 79-85.

91. Miller B, Marshall JC. Coercive sex on the university campus. *Journal of College Student Personnel* 1987.

92. Minow JC, Einolf CJ. Sorority participation and sexual assault risk. *Violence against women* 2009; **15**(7): 835-51.

93. Moeller LM. The prevalence of sexual assault among sorority women at California State University, Long Beach: California State University, Long Beach; 1996.

94. Mohler-Kuo M, Dowdall GW, Koss MP, Wechsler H. Correlates of rape while intoxicated in a national sample of college women. *Journal of studies on alcohol* 2004; **65**(1): 37-45.

95. Moreno-Cubillos CL, Sepúlveda-Gallego LE, Restrepo-Rendon LF. Prevalencia de violencia y discriminación contra la mujer en la Facultad de Ciencias para la Salud, Universidad de Caldas, Colombia, 2010-2011. *Revista colombiana de obstetricia y ginecología* 2013; **64**(1): 12-20.

96. Nasta A, Shah B, Brahmanandam S, et al. Sexual victimization: Incidence, knowledge and resource use among a population of college women. *Journal of pediatric and adolescent gynecology* 2005; **18**(2): 91-6.

97. Navarro JN, Clevenger S. Investigating students' experiences with sexual victimization at a rural institution. *Contemporary Rural Social Work* 2016; **8**(2): 18-36.

98. Neilson EC, Gilmore AK, Pinsky HT, Shepard ME, Lewis MA, George WH. The use of drinking and sexual assault protective behavioral strategies: Associations with sexual victimization and revictimization among college women. *Journal of interpersonal violence* 2018; **33**(1): 137-58.

99. Newton-Taylor BG, David. Prevalence and factors associated with physical and sexual assault of female university students in Ontario. *Health care for women international* 1998; **19**(2): 155-64.

100. Palmer JE, Perrotti C. Measuring self-reported sexual victimization experiences at one university: A comparison of methods. *Journal of Student Affairs Research and Practice* 2016; **53**(4): 403-15.

101. Palmer RS, McMahon TJ, Rounsaville BJ, Ball SA. Coercive sexual experiences, protective behavioral strategies, alcohol expectancies and consumption among male and female college students. *Journal of interpersonal violence* 2010; **25**(9): 1563-78.

102. Parr NJ. Sexual assault and co-occurrence of mental health outcomes among cisgender female, cisgender male, and gender minority US college students. *Journal of Adolescent Health* 2020; **67**(5): 722-6.

103. Patton W, Mannison M. Sexual coercion in dating situations among university students: Preliminary Australian data. *Australian Journal of Psychology* 1995; **47**(2): 66-72.

104. Phipps A, Smith G. Violence against women students in the UK: Time to take action. *Gender and Education* 2012; **24**(4): 357-73.

105. Ray CM, Tyler KA, Gordon Simons L. Risk factors for forced, incapacitated, and coercive sexual victimization among sexual minority and heterosexual male and female college students. *Journal of Interpersonal Violence* 2021; **36**(5-6): 2241-61.

106. Richardson HB, Armstrong JL, Hines DA, Reed KMP. Sexual violence and help-seeking among LGBQ and heterosexual college students. *Partner Abuse* 2015; **6**(1): 29-46.

107. Roberts N, Donovan C, Durey M. Gendered landscapes of safety: How women construct and navigate the urban landscape to avoid sexual violence. *Criminology & Criminal Justice* 2022; **22**(2): 287-303.

108. Rogers DL, Calderón Galassi ML, Espinosa JC, et al. Nonchildhood sexual abuse in Mexican American and Mexican college students. *Journal of Aggression, Maltreatment & Trauma* 2017; **26**(2): 191-210.

109. Russell A. Sorority Women and Sexual Assault: A Feminist Routine Activities Framework: The George Washington University; 2018.

110. Saldarriaga L, Rocha C, Castro D, Jiménez-Moya G, Carvacho H, Bohner G. Sexual violence victimization among undergraduates at a Chilean university. *International Journal of Conflict and Violence (IJCV)* 2020; **14**: 1-14.

111. Santelli JS, Grilo SA, Choo T-H, et al. Does sex education before college protect students from sexual assault in college? *PloS one* 2018; **13**(11): e0205951.

112. Scholl JA, Cogan C, Micol RL, Steward J, Hancock K, Davis JL. Physical and sexual violence on college campuses: Considerations for international students. *Journal of American college health* 2021; **69**(3): 331-4.

113. Schuster I, Krahé B, Toplu-Demirtaş E. Prevalence of sexual aggression victimization and perpetration in a sample of female and male college students in Turkey. *The Journal of Sex Research* 2016; **53**(9): 1139-52.

114. Seabrook RC, McMahon S, Duquaine BC, Johnson L, DeSilva A. Sexual assault victimization and perceptions of university climate among bisexual women. *Journal of Bisexuality* 2018; **18**(4): 425-45.

115. Silbert KN. Negative Outcomes Associated with Childhood Sexual Abuse. 2018.

116. Sivertsen B, Nielsen MB, Madsen IE, Knapstad M, Lønning KJ, Hysing M. Sexual harassment and assault among university students in Norway: a cross-sectional prevalence study. *BMJ open* 2019; **9**(6): e026993.

117. Sriwongtong M, Nattiv A, Florczyk D, Sim M, Kirschner M. Sexual abuse of female collegiate athletes compared to non-athletes. *Clinical Journal of Sport Medicine* 2019; **29**(2): 162-71.

118. Steele B, Degli Esposti M, Mandeville P, Hamnett G, Nye E, Humphreys DK. Sexual violence among students attending a higher education institution in the UK (OUR SPACE): a cross-sectional survey. *The Lancet* 2021; **398**: S83.

119. Stephens SG. Relationship of sexual violence and high-risk behaviors among male and female US college students: The Florida State University; 2016.

120. Stepleton K, McMahon S, Potter CC, MacKenzie MJ. Prior sexual victimization and disclosure of campus sexual violence among college students. *Journal of College Counseling* 2019; **22**(1): 56-69.

121. Stoner JE. Sexual Violence Victimization, Mental Health, and University-Based Health Service Use Among College Females: Old Dominion University; 2018.

122. Tora A. Assessment of sexual violence against female students in Wolaita Sodo University, Southern Ethiopia. *Journal of interpersonal violence* 2013; **28**(11): 2351-67.

123. Vanderwoerd JR, Cheng A. Sexual Violence on Religious Campuses. *Canadian Journal of Higher Education* 2017; **47**(2): 1-21.

124. Walsh WA, Banyard VL, Moynihan MM, Ward S, Cohn ES. Disclosure and service use on a college campus after an unwanted sexual experience. *Journal of Trauma & Dissociation* 2010; **11**(2): 134-51.

125. Wang C, Dong X, Yang J, et al. Sexual violence experienced by male and female Chinese college students in Guangzhou. *Injury prevention* 2015; **21**(e1): e99-e104.

126. Ward SK, Chapman K, Cohn E, White S, Williams K. Acquaintance rape and the college social scene. *Family Relations* 1991: 65-71.

127. White CN. Comparison of intimate partner violence and sexual assault among Black and White college women at Predominantly White Institutions (PWIS): University of South Carolina; 2017.

128. Wigderson S, Katz J. Feminine ideology and sexual assault: Are more traditional college women at greater risk? *Violence Against Women* 2015; **21**(5): 616-31.

129. Wiscombe KJ. An exploratory analysis of sexual violence and rape myth acceptance at a small liberal arts university: University of Kansas; 2012.

130. Zamudio Sánchez FJ, Andrade Barrera MA, Arana Ovalle RI, Alvarado Segura AA. Violencia de género sobre estudiantes universitarios (as). *Convergencia* 2017; **24**(75): 133-57.

131. Zotareli V, Faúndes A, Osis MJD, Duarte GA, Sousa MHd. Gender and sexual violence among students at a Brazilian university. *Revista Brasileira de Saúde Materno Infantil* 2012; **12**: 37-46.
