# Supplementary material for: The Prevalence of Sexual Assault Among Higher Education Students: A Systematic Review With Meta-Analyses
Source: Trauma Violence Abuse. 2023 Sep 20;25(3):1885–98. doi: 10.1177/15248380231196119 (PMC11155219; doi:10.1177/15248380231196119)
Supplement: sj-docx-4-tva-10.1177_15248380231196119 – Supplemental material for The Prevalence of Sexual Assault Among Higher Education Students: A Systematic Review With Meta-Analyses [file sj-docx-4-tva-10.1177_15248380231196119.docx]

| **Author** | **Date** | **Was the sample frame appropriate for target population?** | **Were study participants sampled in an appropriate way?** | **Was the sample size adequate?** | **Were the study subjects and setting described in detail?** | **Was data analysis conducted with sufficient coverage of the sample?** | **Were valid methods used for the identification of the condition?** | **Was the condition measured in a standard, reliable way?** | **Was there appropriate statistical analysis?** | **Was the response rate adequate?** | **Overall score (/9)** |
| --- | --- | --- | --- | --- | --- | --- | --- | --- | --- | --- | --- |
| Adejimi | 2016 | 1 | 1 | 1 | 1 | 1 | NR | 1 | 1 | NR | 7 |
| Adhia | 2022 | 1 | 1 | 1 | 1 | 1 | 1 | 0 | 1 | 0 | 7 |
| Adinew | 2017 | 1 | 0 | 1 | 1 | 0 | 1 | 1 | 1 | 1 | 7 |
| Ajayi | 2021 | 1 | 1 | 1 | 1 | 1 | 0 | 0 | 1 | 1 | 7 |
| Ameral | 2020 | 1 | 1 | 1 | 1 | 1 | 1 | 0 | 1 | 0 | 7 |
| Amos | 2008 | 1 | 1 | 0 | 1 | 1 | NR | 1 | 1 | NR | 6 |
| Andar | 2014 | 1 | 1 | 0 | 1 | 1 | 0 | 1 | 1 | NR | 6 |
| Anderson | 2018 | 1 | 1 | 1 | 1 | 1 | 1 | 1 | 1 | NR | 8 |
| Anthony | 2012 | 1 | 0 | 0 | 1 | 1 | 1 | 1 | 1 | NR | 6 |
| Atkinson | 2020 | 1 | 0 | 0 | 1 | 0 | 1 | 1 | 0 | NR | 4 |
| Backhaus | 2019 | 1 | 1 | 1 | 1 | 1 | 0 | 1 | 1 | NR | 7 |
| Banyard | 2017 | 1 | 1 | 1 | 1 | 1 | 0 | 1 | 1 | NR | 7 |
| Banyard | 2007 | 1 | 1 | 0 | 1 | 1 | 0 | 1 | 1 | 0 | 6 |
| Banyard | 2020 | 1 | 0 | 1 | 0 | 1 | 1 | 0 | 1 | 0 | 5 |
| Barrick | 2012 | 1 | 1 | 1 | 1 | 1 | NR | 1 | 1 | 0 | 7 |
| Bell | 2015 | 1 | 1 | 1 | 1 | 1 | NR | 1 | 1 | 0 | 7 |
| Beres | 2020 | 1 | NR | 1 | NR | 1 | 1 | 1 | NR | 0 | 5 |
| Bergeron | 2016 | 1 | 1 | 1 | 1 | 1 | 1 | 1 | 1 | 1 | 9 |
| Bird | 2016 | 1 | 0 | 1 | 1 | 0 | 1 | 1 | 1 | NR | 6 |
| Blanco | 2021 | 1 | 1 | 1 | 1 | 1 | 1 | 1 | 1 | 1 | 9 |
| Bryant | 2000 | 1 | 0 | 1 | 1 | 0 | 1 | 1 | 1 | 1 | 7 |
| Cantor | 2015 | 1 | 1 | 1 | 1 | 1 | NR | 1 | 1 | 0 | 7 |
| Cass | 2007 | 1 | NR | 1 | 1 | NR | NR | 1 | 1 | NR | 5 |
| Castaño-Castrillón | 2010 | 1 | 0 | 0 | 1 | 1 | 0 | 1 | 1 | NR | 5 |
| Chang Wang | 2015 | 1 | 0 | 1 | 1 | 0 | NR | 1 | 1 | 1 | 6 |
| Chen | 1996 | 1 | NR | 1 | 1 | NR | NR | 1 | 1 | NR | 5 |
| Chen | 2021 | 1 | 1 | 1 | 1 | 1 | 1 | 1 | 1 | NR | 8 |
| Choi | 2018 | 1 | 0 | 0 | 1 | 0 | 1 | 1 | 1 | NR | 5 |
| Copenhaver | 1991 | 1 | 0 | 0 | 1 | 1 | 1 | 1 | 1 | 0 | 6 |
| Coulter | 2017 | 1 | 0 | 1 | 1 | 1 | NR | 1 | 1 | NR | 6 |
| Coulter | 2020 | 1 | 0 | 1 | 1 | 1 | 0 | 0 | 1 | NR | 5 |
| Curtis | 2011 | 1 | 0 | 1 | 1 | 0 | 1 | 1 | 1 | NR | 6 |
| Cusano | 2021 | 1 | 1 | 1 | 1 | 1 | 1 | 1 | 1 | 0 | 8 |
| Daigle | 2019 | 1 | 1 | 1 | 1 | 1 | 0 | 1 | 1 | NR | 7 |
| Daigle | 2008 | 1 | 1 | 1 | 1 | 1 | 1 | 1 | 1 | NR | 8 |
| Dasgupta | 2020 | 1 | NR | 1 | 0 | NR | NR | NR | NR | NR | 2 |
| DeKeseredy | 2019 | 1 | 0 | 1 | 1 | 0 | 1 | 1 | 1 | NR | 6 |
| DeKeseredy | 1993 | 1 | 1 | 0 | 1 | 0 | 1 | 1 | 1 | NR | 6 |
| Dion | 2021 | 1 | 1 | 1 | 0 | 1 | 1 | 1 | 1 | NR | 7 |
| Echeverrìa | 2017 | 1 | 0 | 1 | 1 | 1 | 0 | 1 | 1 | NR | 6 |
| Edwards | 2015 | 1 | 1 | 1 | 1 | 1 | NR | 1 | 1 | 0 | 7 |
| Eisenberg | 2017 | 1 | 0 | 1 | 1 | 1 | NR | 1 | 1 | 0 | 6 |
| Fielding-Miller | 2019 | 1 | 0 | 0 | 1 | 1 | 1 | 1 | 1 | 0 | 6 |
| Finkelson | 1995 | 1 | 0 | 0 | 1 | 1 | NR | 1 | 1 | 1 | 6 |
| Finley | 1993 | 1 | 1 | 0 | 1 | 0 | 1 | 1 | 1 | 1 | 7 |
| Fisher | 2003 | 1 | 0 | 1 | 1 | 1 | NR | 1 | 1 | 1 | 7 |
| Fisher* | 1999 | 1 | 0 | 1 | 1 | 1 | NR | 1 | 1 | 1 | 7 |
| Flack | 2015 | 1 | 1 | 0 | 1 | 1 | 1 | 1 | 1 | 1 | 8 |
| Flack | 2015 | 1 | 1 | 0 | 1 | 1 | 1 | 1 | 1 | 1 | 8 |
| Flack | 2007 | 1 | 1 | 0 | 1 | 1 | NR | 1 | 1 | 1 | 7 |
| Ford | 2016 | 1 | 0 | 1 | 1 | 0 | NR | 1 | 1 | NR | 5 |
| Forke | 2018 | 1 | 0 | 1 | 1 | 1 | NR | 1 | 1 | 1 | 7 |
| Fuentes-Pumarola | 2021 | 1 | 0 | 1 | 1 | 1 | 1 | 1 | 0 | NR | 6 |
| Gartner | 2019 | 1 | 0 | 0 | 1 | 1 | 1 | 1 | 1 | NR | 6 |
| Gross | 2006 | 1 | 0 | 1 | 1 | 0 | 1 | 1 | 1 | NR | 6 |
| Haughey | 2018 | 1 | 1 | 1 | 1 | 1 | NR | 1 | 1 | NR | 7 |
| Herbenick | 2019 | 1 | 1 | 1 | 1 | 1 | 1 | 1 | 1 | 0 | 8 |
| Herres | 2021 | 1 | 1 | 1 | 0 | 1 | 1 | 1 | 0 | 0 | 6 |
| Hines | 2012 | 1 | 1 | 1 | 1 | 1 | 1 | 1 | 1 | 0 | 8 |
| Holland | 2020 | 1 | 1 | 1 | 1 | 1 | 1 | 1 | 1 | 1 | 9 |
| Holloway | 2018 | 1 | 1 | 1 | 1 | 1 | 1 | 1 | 1 | 0 | 8 |
| Hossain | 2014 | 1 | 1 | 1 | 1 | 1 | NR | 1 | 1 | NR | 7 |
| Howard | 2008 | 1 | 0 | 0 | 1 | 0 | NR | 1 | 1 | 0 | 4 |
| Hoxmeier | 2016 | 1 | 1 | 1 | 1 | 1 | NR | 1 | 1 | NR | 7 |
| Iliyasu | 2011 | 1 | 0 | 0 | 1 | 1 | 1 | 1 | 1 | 1 | 7 |
| Johns | 2001 | 1 | 1 | 1 | 1 | 1 | 1 | 1 | 1 | 0 | 8 |
| Johnson | 2016 | 1 | 0 | 0 | 1 | 1 | 0 | 1 | 1 | NR | 5 |
| Jordan-Simmons | 2001 | 1 | 0 | 0 | 1 | 0 | 1 | 1 | 1 | NR | 5 |
| Jouriles | 2020 | 1 | 0 | 1 | 1 | 1 | 1 | 1 | 1 | 0 | 7 |
| Kammer-Kerwick | 2019 | 1 | 1 | 1 | 1 | 1 | 1 | 1 | 1 | 0 | 8 |
| Kilpatrick | 2007 | 1 | 1 | 1 | 1 | 1 | NR | 1 | 1 | NR | 7 |
| Kimble | 2008 | 1 | 0 | 0 | 1 | 1 | 1 | 1 | 1 | 1 | 7 |
| Kirkner | 2020 | 1 | 1 | 1 | 1 | 1 | 1 | 1 | 1 | 0 | 8 |
| Krebs | 2009 | 1 | 1 | 1 | 1 | 1 | NR | 1 | 1 | 0 | 7 |
| Krebs | 2011 | 1 | 1 | 1 | 1 | 1 | NR | 1 | 1 | 0 | 7 |
| Kullima | 2010 | 1 | 0 | 0 | 1 | 1 | NR | 1 | 1 | 1 | 6 |
| Leone | 2016 | 1 | NR | 0 | 1 | NR | NR | 1 | 1 | NR | 4 |
| Lindquist* | 2013 | 1 | 1 | 1 | 1 | 1 | NR | 1 | 1 | 0 | 7 |
| Lott | 1982 | 1 | NR | 1 | 1 | NR | NR | 1 | 1 | 0 | 5 |
| Luetke | 2020 | 1 | 1 | 0 | 1 | 1 | NR | 1 | 1 | NR | 6 |
| Lydston | 2016 | 1 | 1 | 1 | 1 | 1 | 1 | 1 | 1 | 0 | 8 |
| Magrin | 2019 | 1 | 0 | 0 | 1 | 0 | 0 | 1 | 1 | NR | 4 |
| Maletsky | 2019 | 1 | 1 | 1 | 1 | 1 | 1 | 1 | 1 | NR | 8 |
| Marcantonio | 2021 | 1 | 0 | 0 | 1 | 1 | 1 | 1 | 0 | NR | 5 |
| Marsil | 2016 | 1 | 0 | 1 | 1 | 0 | 1 | 1 | 1 | NR | 6 |
| Martin | 2005 | 1 | 0 | 0 | 1 | 1 | 0 | 1 | 1 | NR | 5 |
| McDougall | 2019 | 1 | 1 | 1 | 1 | 1 | NR | 1 | 1 | 0 | 7 |
| McMahon | 2018 | 1 | 1 | 1 | 1 | 1 | 1 | 1 | 1 | NR | 8 |
| Mellins | 2017 | 1 | 0 | 1 | 1 | NR | 1 | 1 | 1 | 1 | 7 |
| Mellins* | 2018 | 1 | 0 | 1 | 1 | NR | 1 | 1 | 1 | 1 | 7 |
| Mennicke | 2021 | 1 | 1 | 1 | 1 | 1 | 1 | 1 | 1 | 0 | 8 |
| Mezie-Okoye | 2014 | 1 | 0 | 0 | 1 | 0 | NR | 1 | 1 | 1 | 5 |
| Miller | 1987 | 1 | 0 | 0 | 1 | 1 | 1 | 1 | 1 | NR | 6 |
| Minow | 2009 | 1 | 0 | 1 | 1 | 0 | 1 | 1 | 1 | 1 | 7 |
| Moeller | 1996 | 1 | 0 | 0 | 1 | 0 | 1 | 1 | 1 | NR | 5 |
| Mohler-Kuo | 2004 | 1 | 1 | 1 | 1 | 1 | NR | 1 | 1 | 1 | 8 |
| Moreno-Cubillos | 2013 | 1 | 0 | 0 | 1 | 1 | 0 | 1 | 1 | NR | 5 |
| Nasta, Aarti | 2005 | 1 | 0 | 0 | 1 | 0 | 1 | 1 | 1 | NR | 5 |
| Navarro | 2016 | 1 | 1 | 0 | 1 | 1 | 1 | 1 | 1 | 0 | 7 |
| Neilson | 2018 | 1 | 0 | 1 | 1 | 0 | 1 | 1 | 1 | NR | 6 |
| Newton-Taylor | 1998 | 1 | 0 | 1 | 1 | 1 | NR | 1 | 1 | 1 | 7 |
| Palmer | 2010 | 1 | 1 | 0 | 1 | 1 | 1 | 1 | 1 | 1 | 8 |
| Palmer | 2016 | 1 | 0 | 0 | 1 | 1 | 1 | 1 | 1 | 1 | 7 |
| Parr | 2020 | 1 | NR | 1 | 1 | NR | NR | 1 | 1 | NR | 5 |
| Patton | 1995 | 1 | 0 | 0 | 1 | 0 | 1 | 1 | 1 | NR | 5 |
| Phipps | 2012 | 1 | 0 | 1 | 1 | 0 | NR | 1 | 1 | NR | 5 |
| Ray | 2018 | 1 | 0 | 1 | 1 | 0 | 1 | 1 | 1 | 1 | 7 |
| Richardson | 2015 | 1 | 1 | 1 | 1 | 1 | 1 | 1 | 1 | 1 | 9 |
| Roberts | 2022 | 1 | 1 | 1 | 0 | 0 | 1 | 0 | 0 | 0 | 4 |
| Rogers | 2017 | 1 | 0 | 0 | 1 | 0 | 1 | 1 | 1 | NR | 5 |
| Russell | 2018 | 1 | NR | 1 | 1 | NR | NR | 1 | 1 | 0 | 5 |
| Saldarriaga | 2020 | 1 | 1 | 1 | 1 | NR | 1 | 1 | 1 | NR | 7 |
| Santelli | 2018 | 1 | 0 | 1 | 1 | 1 | 1 | 1 | 1 | 1 | 8 |
| Scholl | 2019 | 1 | 1 | 1 | 1 | 1 | 1 | 1 | 1 | NR | 8 |
| Scholl | 2021 | 1 | NR | 0 | 0 | 0 | 1 | 1 | 0 | NR | 3 |
| Schuster | 2016 | 1 | 1 | 1 | 1 | 1 | 1 | 1 | 1 | NR | 8 |
| Seabrook | 2019 | 1 | 1 | 1 | 1 | 1 | 1 | 1 | 1 | 0 | 8 |
| Silbert | 2018 | 1 | 0 | 0 | 1 | 0 | 1 | 1 | 1 | NR | 5 |
| Sivertsen | 2019 | 1 | 1 | 1 | 1 | 1 | 1 | 1 | 1 | 0 | 8 |
| Sriwongtong | 2019 | 1 | 0 | 0 | 1 | NR | 1 | 1 | 1 | NR | 5 |
| Steele | 2021 | 1 | 1 | 0 | 1 | 1 | 1 | 1 | 1 | 1 | 8 |
| Stephens | 2016 | 1 | 0 | 1 | 1 | 1 | NR | 1 | 1 | NR | 6 |
| Stepleton | 2019 | 1 | 1 | 1 | 1 | 1 | 1 | 1 | 1 | 0 | 8 |
| Stoner | 2019 | 1 | 0 | 0 | 1 | 0 | 1 | 1 | 1 | NR | 5 |
| Tora | 2013 | 1 | 0 | 0 | 1 | 1 | NR | 1 | 1 | 1 | 6 |
| Vanderwoerd | 2017 | 1 | 1 | 0 | 1 | 1 | 1 | 1 | 1 | 0 | 7 |
| Walsh | 2010 | 1 | 0 | 1 | 1 | 1 | NR | 1 | 1 | 1 | 7 |
| Wang | 2015 | 1 | 0 | 1 | 1 | 0 | NR | 1 | 1 | 1 | 6 |
| Ward | 1991 | 1 | 0 | 1 | 1 | 1 | NR | 1 | 1 | NR | 6 |
| White | 2017 | 1 | 0 | 1 | 1 | 1 | 1 | 1 | 1 | NR | 7 |
| Wigderson | 2015 | 1 | 0 | 0 | 1 | 0 | 1 | 1 | 1 | NR | 5 |
| Wiscombe | 2012 | 1 | 0 | 0 | 1 | 0 | 1 | 1 | 1 | NR | 5 |
| Zamudio-Sanchez | 2017 | 1 | 1 | 0 | 1 | 1 | 0 | 1 | 1 | NR | 6 |
| Zotareli | 2012 | 1 | 1 | 1 | 1 | 1 | 1 | 1 | 1 | 0 | 8 |
