# Supplementary material for: The Prevalence of Sexual Assault Among Higher Education Students: A Systematic Review With Meta-Analyses
Source: Trauma Violence Abuse. 2023 Sep 20;25(3):1885–98. doi: 10.1177/15248380231196119 (PMC11155219; doi:10.1177/15248380231196119)
Supplement: sj-docx-5-tva-10.1177_15248380231196119 – Supplemental material for The Prevalence of Sexual Assault Among Higher Education Students: A Systematic Review With Meta-Analyses [file sj-docx-5-tva-10.1177_15248380231196119.docx]

Figure 1. Sexual assault prevalence for women in the Region of the Americas


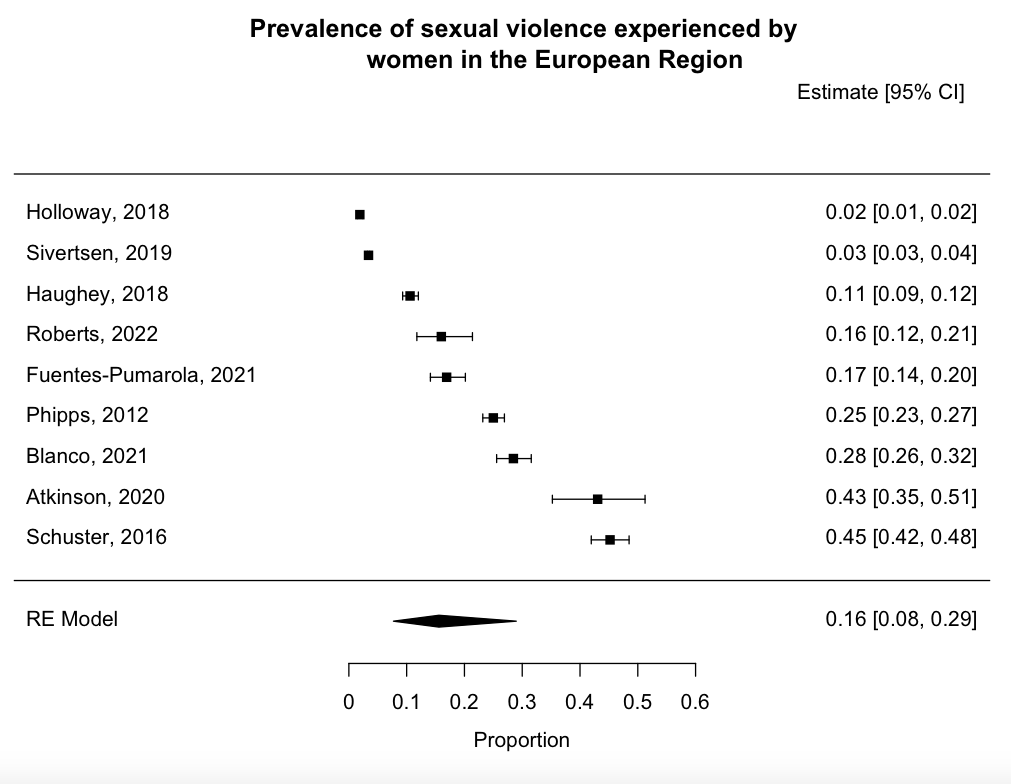


Figure 2. Sexual assault prevalence for women in the European Region

Figure 3. Sexual assault prevalence for women in the African Region


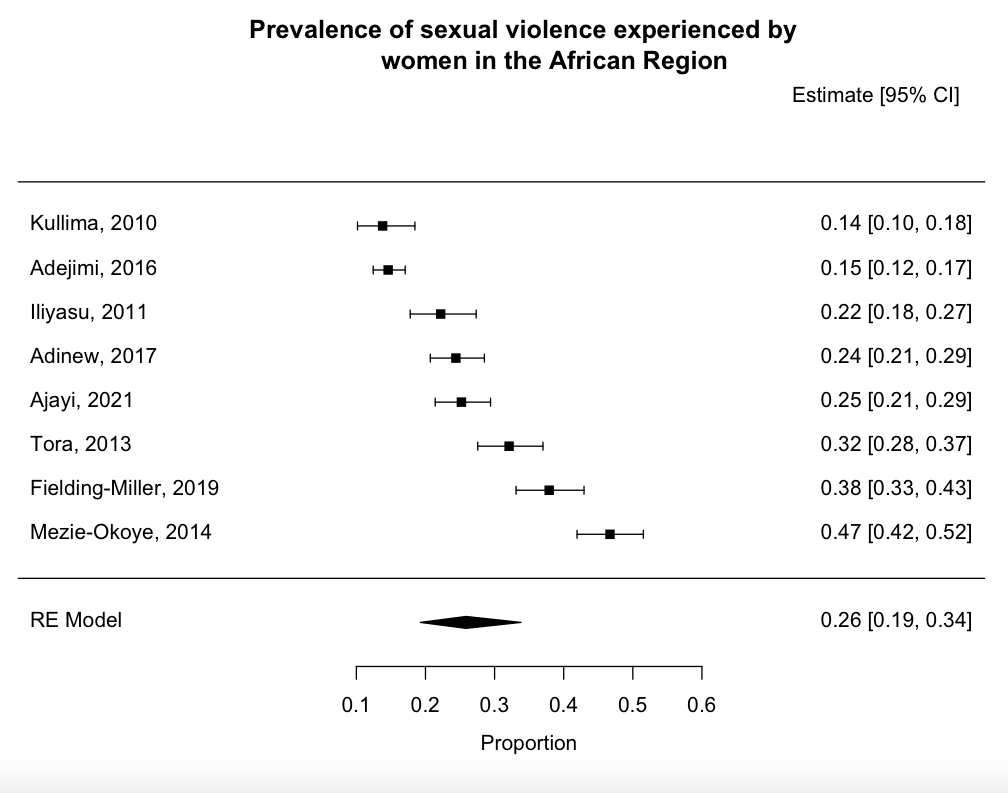


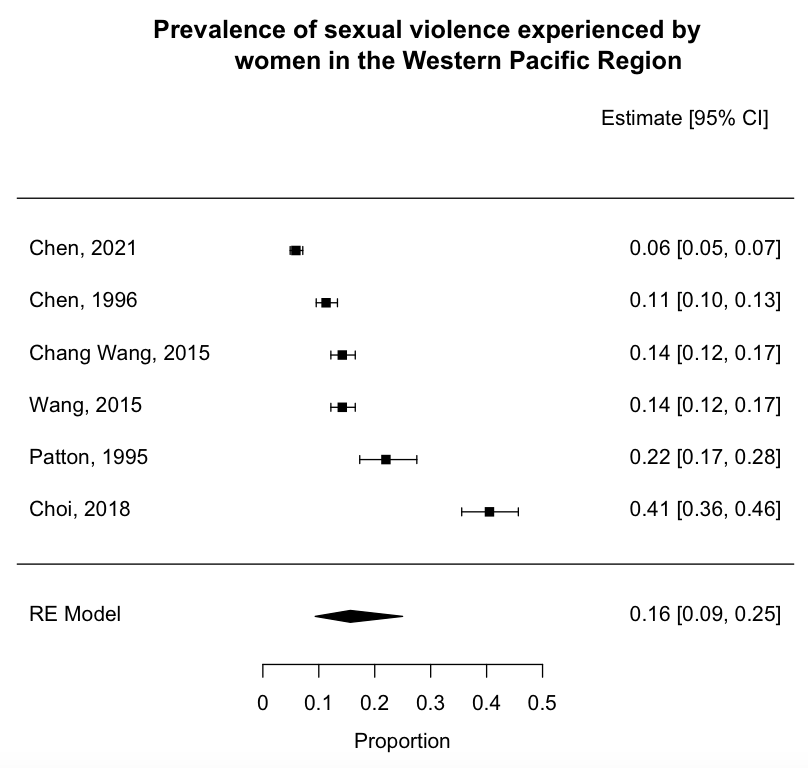


Figure 4. Sexual assault prevalence for women in the Western Pacific Region

Figure 5. Sexual assault prevalence for men in the Region of the Americas


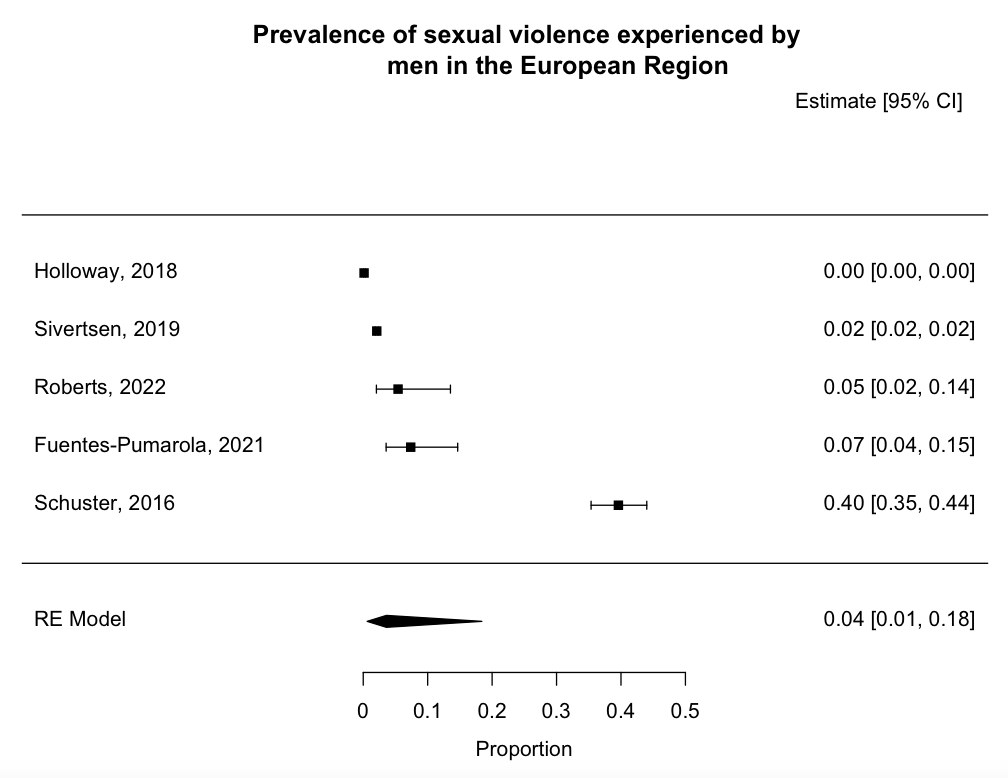

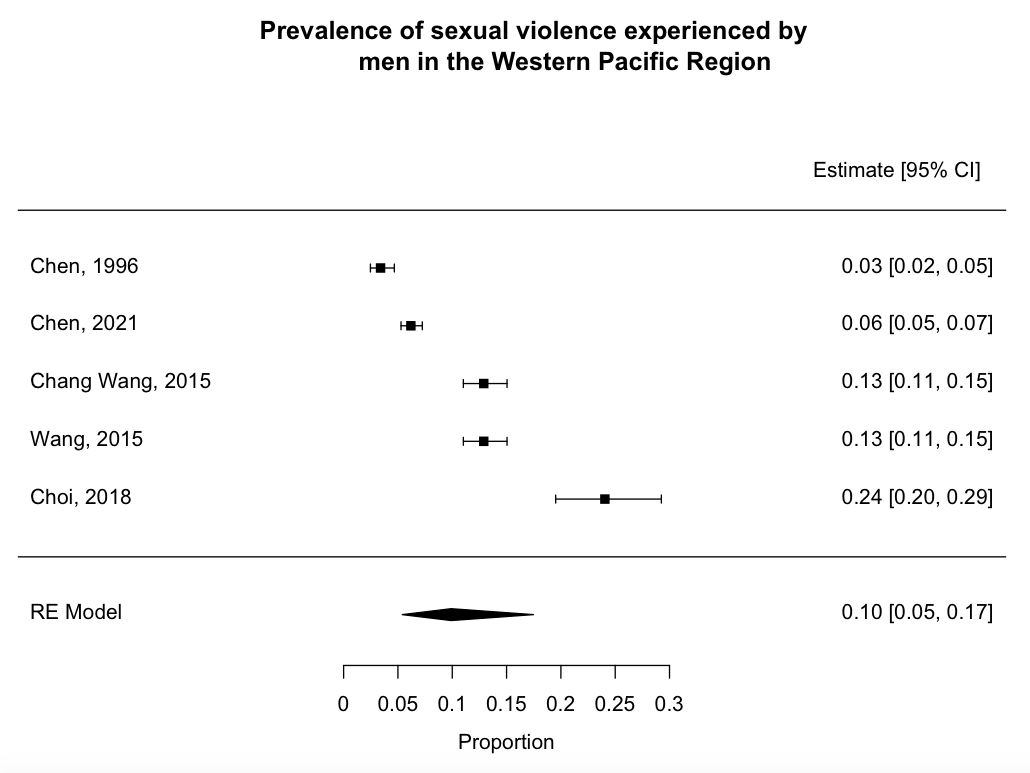


Figure 7. Sexual assault prevalence for men in the Western Pacific Region

Figure 6. Sexual assault prevalence for men in the European Region
