# Supplementary material for: The Prevalence of Sexual Assault Among Higher Education Students: A Systematic Review With Meta-Analyses
Source: Trauma Violence Abuse. 2023 Sep 20;25(3):1885–98. doi: 10.1177/15248380231196119 (PMC11155219; doi:10.1177/15248380231196119)
Supplement: sj-docx-6-tva-10.1177_15248380231196119 – Supplemental material for The Prevalence of Sexual Assault Among Higher Education Students: A Systematic Review With Meta-Analyses [file sj-docx-6-tva-10.1177_15248380231196119.docx]

**Women**

| **Author** | **Date** | **Country** | **N** | **Prevalence of SV** | **Construct measured** | **Tool** | **Recall period** |
| --- | --- | --- | --- | --- | --- | --- | --- |
| Adejimi | 2016 | Nigeria | 898 | 0.15 | Sexual assault | NR | Past year (12 months) |
| Adhia | 2022 | United States | 679 | 0.36 | Sexual violence | American College Health Association, 2021 and Centers for Disease Control and Prevention, 2007 | Entire time at HEI |
| Adinew | 2017 | Ethiopia | 462 | 0.24 | Sexual violence | WHO multicounty study on women’s health and life events | Past academic year |
| Ajayi | 2021 | South Africa | 451 | 0.252 | Sexual violence | Author developed questions | Past year |
| Andar | 2014 | United States | 233 | 0.44 | Sexual assault | College Sexual Assault Survey | Entire time at HEI |
| Anthony | 2012 | United States | 258 | 0.36 | Sexual violence | Sexual Experience Survey | Past year (12 months) |
| Atkinson | 2020 | United Kingdom | 144 | 0.43 | Sexual violence | AAU Campus Climate Survey (Cantor et al., 2015) | Entire time at HEI |
| Banyard | 2007 | United States | 408 | 0.20 | Unwanted sexual contact | Ward, Chapman, Cohn, White, & Williams, 1991 | Past 6 months |
| Barrick | 2012 | United States | 3951 | 0.10 | Sexual assault | NR | Entire time at HEI |
| Bell | 2015 | United States | 463 | 0.16 | Rape | NR | Entire time at HEI |
| Bergeron | 2016 | Canada | 6611 | 0.21 | Sexual violence | Sexual Experiences Questionnaire (French) | Entire time at HEI |
| Bird | 2016 | United States | 603 | 0.08 | Sexual assault | Sexual Experience Survey | Entire time at HEI |
| Blanco | 2021 | Spain | 871 | 0.29 | Sexual violence | SES | Past year |
| Bryant | 2000 | United States | 493 | 0.11 | Sexual violence | Sexual Experience Survey | Past 3 months |
| Cantor | 2015 | United States | 3,347 | 0.13 | Sexual violence | NR | Entire time at HEI |
| Cass | 2007 | United States | 3010 | 0.03 | Sexual assault | NR | Entire time at HEI |
| Chang Wang | 2015 | China | 988 | 0.14 | Sexual violence | NR | Past year (12 months) |
| Chen | 2021 | China | 1692 | 0.59 | Sexual abuse | Sexual Assault Scale (Chen et al., 2006) | Entire time at HEI |
| Chen | 1996 | Taiwan | 1074 | 0.11 | Sexual abuse | NR | Entire time at HEI |
| Choi | 2018 | China | 359 | 0.41 | Sexual violence | Conflict Tactics Scale | Past year (12 months) |
| Copenhaver | 1991 | United States | 140 | 0.42 | Sexual violence | Sexual Experience Survey | Entire time at HEI |
| Coulter | 2017 | United States | 48308 | 0.09 | Sexual assault | NR | Past year (12 months) |
| Curtis | 2011 | United States | 586 | 0.16 | Sexual violence | Sexual Experience Survey | Past year (12 months) |
| Cusano | 2021 | United States | 1534 | 0.25 | Sexual violence | Campus Sexual Assault study | Entire time at HEI |
| Daigle | 2008 | United States | 4446 | 0.16 | Sexual violence | Sexual Experience Survey | Past academic year |
| Dasgupta | 2020 | India | 567 | 0.1 | Sexual violence | NR | Entire time at HEI |
| DeKeseredy | 2019 | United States | 2960 | 0.34 | Sexual assault | Sexual Experience Survey | Entire time at HEI |
| DeKeseredy | 1993 | Canada | 219 | 0.33 | Sexual assault | Sexual Experience Survey | Past year (12 months) |
| Echeverrìa | 2017 | Mexico | 1176 | 0.03 | Sexual violence | NR | Entire time at HEI |
| Edwards | 2015 | United States | 3974 | 0.16 | Sexual assault | NR | Past 6 months |
| Eisenberg | 2017 | United States | 7308 | 0.07 | Sexual assault | NR | Past year (12 months) |
| Fielding-Miller | 2019 | Eswatini | 372 | 0.38 | Completed sexual assault | Sexual Experience Survey | Past year (12 months) |
| Finkelson | 1995 | United States | 140 | 0.05 | Date rape | NR | Entire time at HEI |
| Finley | 1993 | United States | 247 | 0.20 | Sexual violence | Sexual Experience Survey | Entire time at HEI |
| Fisher | 2003 | United States | 4446 | 0.14 | Sexual violence | NR | Past academic year |
| Flack | 2015 | United States | 373 | 0.44 | Sexual assault | Sexual Experience Survey | Entire time at HEI |
| Flack | 2015 | United States | 208 | 0.19 | Sexual violence | Sexual Experience Survey | Past 6 months |
| Flack | 2007 | United States | 107 | 0.23 | Unwanted sex | NR | Entire time at HEI |
| Ford | 2016 | United States | 14604 | 0.25 | Sexual assault | NR | Entire time at HEI |
| Forke | 2018 | United States | 520 | 0.16 | Sexual violence | NR | Entire time at HEI |
| Fuentes-Pumarola | 2021 | Spain | 591 | 0.17 | Sexual Violence | Sexual Experience Survey | Entire time at HEI |
| Gartner | 2019 | United States | 220 | 0.38 | Sexual assault | Sexual Experience Survey | Past year (12 months) |
| Gross | 2006 | United States | 903 | 0.27 | Unwanted sex | Washington State University Sexual Assault Task Force | Entire time at HEI |
| Haughey | 2018 | Northern Ireland | 1976 | 0.11 | Attempted penetrative assault | NR | Entire time at HEI |
| Herbenick | 2019 | United States | 3,579 | 0.16 | Non-consensual sex | The National Women’s Study and National College Women Sexual Victimization study | Entire time at HEI |
| Hines | 2012 | United States | 1381 | 0.07 | Sexual assault | University of New Hampshire (Banyard et al., 2007). | Past academic year |
| Holland | 2020 | United States | 840 | 0.34 | Sexual assault | Sexual Experience Survey | Entire time at HEI |
| Holloway | 2018 | Wales | 3,364 | 0.02 | Alcohol-involved rape | Core Institute national survey questionnaire 2012 | Past academic year |
| Hossain | 2014 | United States | 10541 | 0.16 | Sexual violence | NR | Past academic year |
| Howard | 2008 | United States | 324 | 0.20 | Alcohol-involved sexual assault | NR | Past 3 months |
| Hoxmeier | 2016 | United States | 12,838 | 0.12 | Sexual violence | NR | Past year (12 months) |
| Iliyasu | 2011 | Nigeria | 291 | 0.22 | Sexual violence | Nigeria Demographic and Health Survey | Entire time at HEI |
| Johns | 2001 | United States | 869 | 0.13 | Sexual assault | Sexual Experience Survey | Entire time at HEI |
| Jordan-Simmons | 2001 | United States | 317 | 0.29 | Sexual violence | Sexual Experience Survey | Entire time at HEI |
| Jouriles | 2020 | United States | 1,008 | 0.24 | Sexual violence | Conflict Tactics Scale | Past 3 months |
| Kammer-Kerwick | 2019 | United States | 10,679 | 0.20 | Sexual violence | Sexual Experience Survey | Entire time at HEI |
| Kilpatrick | 2007 | United States | 2000 | 0.13 | Rape | NR | Past year (12 months) |
| Kimble | 2008 | United States | 102 | 0.41 | Unwanted sex | Sexual Experience Survey | Past year (12 months) |
| Krebs | 2009 | United States | 5446 | 0.19 | Sexual assault | NR | Past year (12 months) |
| Krebs | 2011 | United States | 3951 | 0.14 | Sexual assault | NR | Entire time at HEI |
| Kullima | 2010 | Nigeria | 268 | 0.14 | Sexual assault | NR | Entire time at HEI |
| Leone | 2016 | United States | 258 | 0.09 | Sexual violence | NR | Past year (12 months) |
| Lott | 1982 | United States | 542 | 0.07 | Sexual assault | NR | Entire time at HEI |
| Lydston | 2016 | United States | 5,825 | 0.13 | Sexual violence | SDSU Sexual Experience Scale | Entire time at HEI |
| Magrin | 2019 | Brazil | 241 | 0.11 | Sexual violence | NR | Past year (12 months) |
| Maletsky | 2019 | United States | 1559 | 0.32 | Sexual violence | Massachusetts Institute of Technology climate study of sexual assault (2014) and White House Task Force toolkit (2014) | Entire time at HEI |
| Marcantonio | 2021 | United States | 551 | 0.48 | Sexual assault | SES | Entire time at HEI |
| Marsil | 2016 | United States | 1150 | 0.11 | Rape | Sexual Experience Survey | Entire time at HEI |
| McDougall | 2019 | Canada | 6939 | 0.07 | Non-consensual sex | NR | Entire time at HEI |
| McMahon | 2018 | United States | 6,082 | 0.07 | Sexual violence | White House Task Force | Entire time at HEI |
| Mellins | 2017 | United States | 928 | 0.28 | Sexual assault | Sexual Experience Survey | Entire time at HEI |
| Mennicke | 2021 | United States | 4881 | 0.07 | Sexual violence | Modified American Association of Universities survey | Past academic year |
| Mezie-Okoye | 2014 | Nigeria | 413 | 0.47 | Sexual violence | NR | Entire time at HEI |
| Miller | 1987 | United States | 472 | 0.14 | Sexual coercion | Sexual Experience Survey | Entire time at HEI |
| Minow | 2009 | United States | 779 | 0.21 | Sexual violence | Adaptation of 1997 National Victimization of College Women Study; 1982 Sexual Experiences Survey | Entire time at HEI |
| Moeller | 1996 | United States | 148 | 0.33 | Sexual assault | Sexual Experience Survey | Entire time at HEI |
| Mohler-Kuo | 2004 | United States | 23980 | 0.05 | Rape | NR | Past academic year |
| Moreno-Cubillos | 2013 | Colombia | 143 | 0.01 | Rape | Ad-hoc survey | Entire time at HEI |
| Nasta, Aarti | 2005 | United States | 234 | 0.38 | Sexual violence | Sexual Experience Survey | Past academic year |
| Navarro | 2016 | United States | 234 | 0.37 | Sexual violence | National Victimization of College Women Study; White House Task Force | Past year (12 months) |
| Neilson | 2018 | United States | 620 | 0.22 | Sexual assault | Sexual Experience Survey | Entire time at HEI |
| Newton-Taylor | 1998 | Canada | 3642 | 0.15 | Sexual assault | NR | Past year (12 months) |
| Palmer | 2010 | United States | 192 | 0.34 | Unwanted sexual contact | Sexual Experience Survey | Past year (12 months) |
| Parr | 2020 | United States | 34,007 | 0.13 | Sexual assault | NR | Past year (12 months) |
| Patton | 1995 | Australia | 253 | 0.22 | Unwillingness to participate in sex | Sexual Experience Survey | Entire time at HEI |
| Phipps | 2012 | United Kingdom | 2058 | 0.25 | Unwanted sex | NR | Entire time at HEI |
| Ray | 2018 | United States | 732 | 0.42 | Sexual violence | Sexual Experience Survey | Past year (12 months) |
| Roberts | 2022 | United Kingdom | 225 | 0.16 | Sexual violence | NUS Hidden Marks | Entire time at HEI |
| Rogers | 2017 | Mexico | 279 | 0.21 | Sexual abuse | Sexual Experience Survey | Past year (12 months) |
| Russell | 2018 | United States | 5446 | 0.34 | Sexual assault | NR | Entire time at HEI |
| Saldarriaga | 2020 | Chile | 1377 | 0.23 | Sexual violence | The Sexual Aggression and Victimization Scale | Past year (12 months) |
| Santelli | 2018 | United States | 954 | 0.14 | Penetrative sexual assault | Sexual Experience Survey | Entire time at HEI |
| Schuster | 2016 | Turkey | 886 | 0.45 | Sexual aggression | The Sexual Aggression and Victimization Scale | Past year (12 months) |
| Seabrook | 2019 | United States | 6067 | 0.17 | Sexual violence | White House Task Force | Entire time at HEI |
| Silbert | 2018 | United States | 157 | 0.49 | Sexual violence | Sexual Experience Survey | Entire time at HEI |
| Sivertsen | 2019 | Norway | 34597 | 0.03 | Rape | Norway Students’ Health and Wellbeing Study | Past year (12 months) |
| Sriwongtong | 2019 | United States | 148 | 0.21 | Sexual assault | Campus Climate Survey (2014-2015) | Entire time at HEI |
| Stephens | 2016 | United States | 64005 | 0.10 | Sexual violence | NR | Past year (12 months) |
| Stepleton | 2019 | United States | 5,242 | 0.14 | Sexual violence | White House Task Force | Entire time at HEI |
| Stoner | 2019 | United States | 454 | 0.14 | Rape | Sexual Experience Survey | Past year (12 months) |
| Tora | 2013 | Ethiopia | 374 | 0.32 | Rape | NR | Past year (12 months) |
| Vanderwoerd | 2017 | Canada | 399 | 0.21 | Unwanted sex | SES | Past year (12 months) |
| Walsh | 2010 | United states | 750 | 0.21 | Unwanted sex contact and intercourse | NR | Past academic year |
| Wang | 2015 | China | 988 | 0.14 | Sexual violence | NR | Past year (12 months) |
| Ward | 1991 | United States | 518 | 0.34 | Unwanted sex | NR | Past semester |
| White | 2017 | United States | 3005 | 0.24 | Sexual assault | National Intimate Partner and Sexual Violence Survey | Past academic year |
| Wigderson | 2015 | United States | 254 | 0.16 | Rape | Sexual Experience Survey | Entire time at HEI |
| Wiscombe | 2012 | United States | 157 | 0.31 | Sexual violence | Sexual Experience Survey | Entire time at HEI |
| Zamudio-Sanchez | 2017 | Mexico | 119 | 0.17 | Sexual Violence | Ad-hoc survey | Entire time at HEI |
| Zotareli | 2012 | Brazil | 1167 | 0.09 | Sexual violence | Conflict Tactics Scale | Entire time at HEI |

**Men**

| **Author** | **Date** | **Country** | **N** | **Prevalence of SV** | **Construct measured** | **Tool** | **Recall period** |
| --- | --- | --- | --- | --- | --- | --- | --- |
| Adejimi | 2016 | Nigeria | 640 | 0.09 | Sexual assault | NR | Past year (12 months) |
| Adhia | 2022 | United States | 317 | 0.12 | Sexual violence | American College Health Association, 2021 and Centers for Disease Control and Prevention, 2007 | Entire time at HEI |
| Amos | 2008 | United States | 181 | 0.09 | Sexual abuse | NR | Past academic year |
| Anderson | 2018 | United States | 396 | 0.17 | Sexual victimisation | Sexual Experience Survey | Past year (12 months) |
| Anthony | 2012 | United States | 190 | 0.35 | Sexual violence | Sexual Experience Survey | Past year (12 months) |
| Banyard | 2007 | United States | 225 | 0.08 | Unwanted sexual contact | Ward, Chapman, Cohn, White, & Williams, 1991 | Past 6 months |
| Bergeron | 2016 | Canada | 2536 | 0.11 | Sexual violence | Sexual Experiences Questionnaire (French) | Entire time at HEI |
| Cantor | 2015 | United States | 2,135 | 0.04 | Sexual violence | NR | Entire time at HEI |
| Cass | 2007 | United States | 462 | 0.01 | Sexual assault | NR | Entire time at HEI |
| Chang Wang | 2015 | China | 1072 | 0.13 | Sexual violence | NR | Past year (12 months) |
| Chen | 2021 | China | 2342 | 0.62 | Sexual abuse | SA scale (Chen et al., 2006) | Entire time at HEI |
| Chen | 1996 | Taiwan | 1073 | 0.03 | Sexual abuse | NR | Entire time at HEI |
| Choi | 2018 | China | 296 | 0.24 | Sexual violence | Conflict Tactics Scale | Past year (12 months) |
| Coulter | 2017 | United States | 22936 | 0.04 | Sexual assault | NR | Past year (12 months) |
| Cusano | 2021 | United States | 709 | 0.06 | Sexual violence | Campus Sexual Assault study | Entire time at HEI |
| Echeverrìa | 2017 | Mexico | 894 | 0.04 | Sexual violence | Ad-hoc survey | Entire time at HEI |
| Edwards | 2015 | United States | 2056 | 0.07 | Sexual assault | NR | Past 6 months |
| Eisenberg | 2017 | United States | 3338 | 0.05 | Sexual assault | NR | Past year (12 months) |
| Finley | 1993 | United States | 278 | 0.23 | Sexual violence | Sexual Experience Survey | Entire time at HEI |
| Flack | 2007 | United States | 71 | 0.18 | Unwanted sex | NR | Entire time at HEI |
| Ford | 2016 | United States | 6581 | 0.13 | Sexual assault | NR | Entire time at HEI |
| Forke | 2018 | United States | 390 | 0.07 | Sexual violence | NR | Entire time at HEI |
| Fuentes-Pumarola | 2021 | Spain | 95 | 0.07 | Sexual violence | SES | Entire time at HEI |
| Herbenick | 2019 | United States | 3,361 | 0.08 | Non-consensual sex | Modified from The National Women’s Study and National College Women Sexual Victimization study | Entire time at HEI |
| Hines | 2012 | United States | 535 | 0.03 | Sexual assault | University of New Hampshire (Banyard et al., 2007). | Past academic year |
| Holloway | 2018 | Wales | 2,749 | 0.00 | Alcohol-involved rape | Core Institute national survey questionnaire (2012) | Past academic year |
| Howard | 2008 | United States | 227 | 0.07 | Alcohol-involved sexual assault | NR | Past 3 months |
| Hoxmeier | 2016 | United States | 6722 | 0.04 | Sexual violence | NR | Past year (12 months) |
| Jouriles | 2020 | United States | 344 | 0.16 | Sexual violence | Conflict Tactics Scale | Past 3 months |
| Kammer-Kerwick | 2019 | United States | 5,912 | 0.09 | Sexual violence | Sexual Experience Survey | Entire time at HEI |
| Lott | 1982 | United States | 377 | 0.01 | Sexual assault | NR | Entire time at HEI |
| Luetke | 2020 | United States | 102 | 0.26 | Sexual violence | NR | Since entering college |
| Lydston | 2016 | United States | 2,991 | 0.03 | Sexual violence | SDSU Sexual Experience Scale | Entire time at HEI |
| Maletsky | 2019 | United States | 807 | 0.11 | Sexual violence | MIT climate study of sexual assault (2014) and White House Task Force toolkit (2014) | Entire time at HEI |
| Marsil | 2016 | United States | 498 | 0.05 | Rape | Sexual Experience Survey | Entire time at HEI |
| McMahon | 2018 | United States | 3,417 | 0.01 | Sexual violence | White House Task Force | Entire time at HEI |
| Mellins | 2017 | United States | 634 | 0.13 | Sexual assault | Sexual Experience Survey | Entire time at HEI |
| Mennicke | 2021 | United States | 3040 | 0.01 | Sexual violence | Modified American Association of Universities survey | Past academic year |
| Miller | 1987 | United States | 323 | 0.15 | Sexual coercion | Sexual Experience Survey | Entire time at HEI |
| Navarro | 2016 | United States | 153 | 0.17 | Sexual violence | National Victimization of College Women Study and White House Task Force (2014) | Past year (12 months) |
| Palmer | 2010 | United States | 178 | 0.31 | Unwanted sexual contact | Sexual Experience Survey | Past year (12 months) |
| Parr | 2020 | United States | 14,908 | 0.04 | Sexual assault | NR | Past year (12 months) |
| Ray | 2018 | United States | 681 | 0.24 | Sexual violence victimisation | Sexual Experience Survey | Past year (12 months) |
| Roberts | 2022 | United Kingdom | 74 | 0.05 | Sexual violence | NUS hidden marks | Entire time at HEI |
| Rogers | 2017 | Mexico | 227 | 0.16 | Sexual abuse | Sexual Experience Survey | Past year (12 months) |
| Saldarriaga | 2020 | Chile | 659 | 0.10 | Sexual violence | The Sexual Aggression and Victimization Scale | Past year (12 months) |
| Santelli | 2018 | United States | 678 | 0.05 | Penetrative sexual assault | Sexual Experience Survey | Entire time at HEI |
| Schuster | 2016 | Turkey | 490 | 0.40 | Sexual aggression | The Sexual Aggression and Victimization Scale | Past year (12 months) |
| Sivertsen | 2019 | Norway | 15457 | 0.02 | Rape | Norway Students’ Health and Wellbeing Study | Past year (12 months) |
| Stephens | 2016 | United States | 32972 | 0.04 | Sexual violence | NR | Past year (12 months) |
| Vanderwoerd | 2017 | Canada | 269 | 0.20 | Unwanted sex | SES | Past year (12 months) |
| Walsh | 2010 | United states | 480 | 0.07 | Unwanted sex contact and sex intercourse | NR | Past academic year |
| Wang | 2015 | China | 1072 | 0.13 | Sexual violence | NR | Past year (12 months) |
| Zamudio-Sanchez | 2017 | Mexico | 88 | 0.11 | Sexual violence | Ad-hoc survey | Entire time at HEI |

**All gender identities**

| **Author** | **Date** | **Country** | **N** | **Prevalence of SV** | **Construct** | **Tool** | **Recall period** |
| --- | --- | --- | --- | --- | --- | --- | --- |
| Adejimi | 2016 | Nigeria | 1538 | 0.12 | Sexual assault | NR | Past year (12 months) |
| Ameral | 2020 | United States | 4710 | 0.05 | Sexual assault | Modified University of New Hampshire survey | Past 2 months |
| Backhaus | 2019 | United States | 60194 | 0.10 | Sexual assault |  | Past year (12 months) |
| Banyard | 2017 | United States | 4275 | 0.12 | Unwanted sexual contact and intercourse | NR | Past 6 months |
| Banyard | 2020 | United States | 5213 | 0.14 | Unwanted contact and intercourse | Banyard et al., 2007; Ward et al., 1991 | Past academic year |
| Beres | 2020 | New Zealand | 1540 | 0.28 | Sexual assault | Administrator-Researcher Campus Climate Consortium tool | Entire time at HEI |
| Bergeron | 2016 | Canada, Quebec | 9284 | 0.18 | Sexual Violence | Sexual Experiences Questionnaire (French) | Entire time at HEI |
| Cass | 2007 | United States | 3036 | 0.04 | Sexual assault | NR | Entire time at HEI |
| Castaño-Castrillón | 2010 | Colombia | 205 | 0.03 | Sexual assault | NR | Entire time at HEI |
| Chang Wang | 2015 | China | 2200 | 0.25 | Sexual violence | NR | Past year (12 months) |
| Chen | 2021 | China | 4034 | 0.06 | Sexual abuse | SA scale (Chen et al., 2006) | Entire time at HEI |
| Choi | 2018 | China | 666 | 0.12 | Sexual violence | Conflict Tactics Scale | Past year (12 months) |
| Coulter | 2017 | United States | 71421 | 0.07 | Sexual assault | NR | Past year (12 months) |
| Daigle | 2019 | United States | 21724 | 0.07 | Sexual violence victimisation | NR | Past year (12 months) |
| Daigle | 2019 | Canada | 22995 | 0.08 | Sexual violence victimisation | NR | Past year (12 months) |
| Dion | 2021 | Canada | 5627 | 0.16 | Unwanted sexual behaviour | SES | Entire time and past 12 months |
| Echeverrìa | 2017 | Mexico | 1949 | 0.03 | Sexual Violence | Ad-hoc survey | Entire time at HEI |
| Edwards | 2015 | United States | 6030 | 0.13 | Sexual assault | NR | Past 6 months |
| Eisenberg | 2017 | United States | 8270 | 0.06 | Sexual assault | NR | Past year (12 months) |
| Flack | 2007 | United States | 178 | 0.17 | Unwanted sex | NR | Entire time at HEI |
| Forke | 2018 | United States | 910 | 0.12 | Sexual violence | NR | Entire time at HEI |
| Fuentes-Pumarola | 2021 | Spain | 686 | 0.16 | Sexual abuse and sexual assault | SES | Entire time at HEI |
| Herres | 2021 | United States | 1611 | 0.10 | Campus sexual violence | SES | Entire time at HEI |
| Holloway | 2018 | Wales | 6113 | 0.01 | Alcohol-involved rape | Core Institute national survey questionnaire (2012) | Past academic year |
| Holloway | 2018 | Wales | 6113 | 0.04 | Alcohol-involved rape | Core Institute national survey questionnaire (2012) | Entire time at HEI |
| Howard | 2008 | United States | 551 | 0.15 | Alcohol-involved sexual assault | NR | Past 3 months |
| Hoxmeier | 2016 | United States | 19581 | 0.09 | Sexual violence victimisation | NR | Past year (12 months) |
| Johnson | 2016 | United States | 19113 | 0.06 | Sexually abusive relationship | NR | Past year (12 months) |
| Kammer-Kerwick | 2019 | United States | 16764 | 0.16 | Sexual violence | Sexual Experience Survey | Entire time at HEI |
| Lydston | 2016 | United States | 8816 | 0.16 | Sexual violence | SDSU Sexual Experience Scale | Entire time at HEI |
| Maletsky | 2019 | United States | 1455 | 0.25 | Sexual violence | MIT climate study of sexual assault (2014) and White House Task Force toolkit (2014) | Entire time at HEI |
| Marsil | 2016 | United States | 1648 | 0.09 | Rape | Sexual Experience Survey | Entire time at HEI |
| Martin | 2005 | United States | 416 | 0.10 | Sexual violence | NR | Past year (12 months) |
| Mellins | 2017 | United States | 1592 | 0.22 | Sexual assault | Sexual Experience Survey | Entire time at HEI |
| Mennicke | 2021 | United States | 8017 | 0.05 | Sexual victimisation | Modified American Association of Universities survey | Past academic year |
| Palmer | 2016 | United States | 365 | 0.15 | Sexual assault | American College Health Association’s National College Health Assessment 2011 | Past year (12 months) |
| Palmer | 2016 | United States | 589 | 0.37 | Sexual assault | Prevention of dating and sexual violence survey 2011 | Past year (12 months) |
| Palmer | 2016 | United States | 590 | 0.15 | Sexual assault | American College Health Association’s National College Health Assessment 2013 | Past year (12 months) |
| Palmer | 2016 | United States | 557 | 0.33 | Sexual assault | Prevention of dating and sexual violence survey 2013 | Past year (12 months) |
| Richardson | 2015 | United States | 2790 | 0.06 | Sexual violence | University of New Hampshire (Banyard et al., 2007). | Past 3 months |
| Saldarriaga | 2020 | Chile | 2046 | 0.19 | Sexual violence | The Sexual Aggression and Victimization Scale | Past year (12 months) |
| Scholl | 2019 | United States | 621 | 0.10 | Sexual violence | White House Task Force | Entire time at HEI |
| Scholl | 2021 | United States | 829 | 0.08 | Sexual violence | White House Task Force | Past year |
| Steele | 2021 | UK | 1608 | 0.18 | Sexual violence | Sexual Experiences Survey | Past year |
| Vanderwoerd | 2017 | Canada | 668 | 0.20 | Unwanted sex | Sexual Experiences Survey | Past year (12 months) |
| Walsh | 2010 | United states | 1230 | 0.16 | Unwanted sex | NR | Past academic year |
| Wang | 2015 | China | 2200 | 0.10 | Sexual violence | NR | Past year (12 months) |

**Other gender identity**

| **Author** | **Date** | **Country** | **N** | **Prevalence of SV** | **Construct measured** | **Tool** | **Recall period** |
| --- | --- | --- | --- | --- | --- | --- | --- |
| Bergeron | 2016 | Canada, Quebec | 6611 | 0.298 | Sexual Violence | Sexual Experiences Questionnaire (French) | Entire time at HEI |
| Cantor | 2015 | United States | 1656 | 0.184 | Sexual violence | NR | Entire time at HEI |
| Coulter | 2020 | United States | 241 | 0.050 | Sexual assault | Author developed questions | Entire time at HEI |
| Coulter | 2017 | United States | 177 | 0.209 | Sexual assault | NR | Past year (12 months) |
| Cusano | 2021 | United States | 28 | 0.214 | Sexual violence | Campus Sexual Assault study | Entire time at HEI |
| Hoxmeier | 2016 | United States | 79 | 0.139 | Sexual violence | NR | Past year (12 months) |
| Kammer-Kerwick | 2019 | United States | 1869 | 0.231 | Sexual violence | Sexual Experience Survey | Entire time at HEI |
| Mennicke | 2021 | United States | 96 | 0.100 | Sexual victimisation | Modified American Association of Universities survey | Past academic year |
| Parr | 2020 | United States | 1523 | 0.180 | Sexual assault | NR | Past year (12 months) |
| Santelli | 2018 | United States | 318 | 0.080 | Penetrative sexual assault | Sexual Experience Survey | Entire time at HEI |
